# Novel type of pilus associated with a Shiga-toxigenic *E. coli* hybrid pathovar conveys aggregative adherence and bacterial virulence

**DOI:** 10.1038/s41426-018-0209-8

**Published:** 2018-12-05

**Authors:** Christina Lang, Angelika Fruth, Gudrun Holland, Michael Laue, Sabrina Mühlen, Petra Dersch, Antje Flieger

**Affiliations:** 1 Division of Enteropathogenic Bacteria and Legionella, Robert Koch Institut, Wernigerode, Saxony-Anhalt 38855 Germany; 2 Division of Advanced Light and Electron Microscopy, Robert Koch Institut, Berlin, 13353 Germany; 3 Department of Molecular Infection Biology, Helmholtz Centre for Infection Research, Braunschweig, Lower Saxony 38124 Germany

## Abstract

A large German outbreak in 2011 was caused by a locus of enterocyte effacement (LEE)-negative enterohemorrhagic *E. coli* (EHEC) strain of the serotype O104:H4. This strain harbors markers that are characteristic of both EHEC and enteroaggregative *E. coli* (EAEC), including aggregative adhesion fimbriae (AAF) genes. Such rare EHEC/EAEC hybrids are highly pathogenic due to their possession of a combination of genes promoting severe toxicity and aggregative adhesion. We previously identified novel EHEC/EAEC hybrids and observed that one strain exhibited aggregative adherence but had no AAF genes. In this study, a genome sequence analysis showed that this strain belongs to the genoserotype O23:H8, MLST ST26, and harbors a 5.2 Mb chromosome and three plasmids. One plasmid carries some EAEC marker genes, such as *aatA* and genes with limited protein homology (11–61%) to those encoding the bundle-forming pilus (BFP) of enteropathogenic *E. coli*. Due to significant protein homology distance to known pili, we designated these as aggregate-forming pili (AFP)-encoding genes and the respective plasmid as pAFP. The *afp* operon was arranged similarly to the operon of BFP genes but contained an additional gene, *afpA2*, which is homologous to *afpA*. The deletion of the *afp* operon, *afpA*, or a nearby gene (*afpR*) encoding an AraC-like regulator, but not *afpA2*, led to a loss of pilin production, piliation, bacterial autoaggregation, and importantly, a >80% reduction in adhesion and cytotoxicity toward epithelial cells. Gene sets similar to the *afp* operon were identified in a variety of *aatA*-positive but AAF-negative intestinal pathogenic *E. coli*. In summary, we characterized widely distributed and novel fimbriae that are essential for aggregative adherence and cytotoxicity in a LEE-negative Shiga-toxigenic hybrid.

## Introduction

A large outbreak in 2011 was caused by an enterohemorrhagic *Escherichia coli* (EHEC) strain of the rare serotype O104:H4, which led to 53 deaths, 855 cases of life-threatening hemolytic uremic syndrome (HUS), and 2,987 cases of gastroenteritis^1,2^. The outbreak-causing strain was shown to be a hybrid that harbors genes characteristic of both EHEC and enteroaggregative *E. coli* (EAEC), and it is therefore referred to as EHEC/EAEC^3,4^. The characterization of such hybrid strains is important because of their high pathogenicity, which is a result of the combined production of Shiga toxin and specific adhesins, the latter of which are encoded by aggregative adherence fimbriae (AAF) genes located on the EAEC virulence plasmid pAA^3,5,6^. Specifically, the pAA of the outbreak strain carries genes encoding type I AAF (AAF/I), the AraC-like regulator AggR, and the antiaggregation protein dispersin (Aap) and its export ABC transporter complex (AatA-AatD), among others^7^. Some genes encoding classical Shiga-toxigenic *E. coli* (STEC)/EHEC virulence determinants such as the intimin-encoding gene *eaeA*, a marker gene for the locus of enterocyte effacement (LEE), the type III-protein secretion system encoding genes within the LEE, and the hemolysin toxin gene (*hlyA*) typically coded on plasmid, were however not found in the outbreak strain^3,4^.

EHEC/EAEC hybrid strains have rarely been reported. Such strains are LEE (*eaeA*)*-*negative, and intimate attachment to host cells by these strains is triggered by other factors, such as AAF. In 1998, a Stx2-producing and *aatA*-positive *E. coli* O111:H2 strain associated with an outbreak of HUS in France was characterized by Morabito et al.^8^. In 1999, a *stx2*- and *aggR*-positive *E. coli* O86:HNM strain was isolated from a 3-year-old child with HUS in Japan^9^. However, whether these strains possessed AAF genes was not reported. Dallman et al. described an HUS case in 2012 that was associated with an *E. coli* O111:H21 isolate expressing *stx2* and carrying pAA with genes encoding AAF/V, dispersin, AggR and the Aat complex^10^. In addition, several O104:H4 EHEC/EAEC strains that predominantly harbored AAF/I genes, although some possessed AAF/III, were described that differed from the 2011 outbreak strain^11–17^.

To further assess the significance of EHEC/EAEC strains in human disease, we previously reanalyzed STEC/EHEC strains from the German National Reference Center (NRC) for *Salmonella* and other Bacterial Enteric Pathogens collected between 2008 and 2012 and identified two strains of further interest. Both strains tested negative for *eaeA* and *hlyA* and positive for *stx2* and the EAEC marker *aatA*, as well as showed aggregative adhesion to HEp-2 cells^18,19^. One of these strains was isolated in 2010 from a case of bloody diarrhea and was shown to belong to the serovar O59:H^−^[19], MLST ST 1136, and encoded genes for type IV AAF (AAF/IV)^19^. The other strain was isolated from a case of diarrhea in 2012 (strain 12-05829) and was observed to be nonmotile with rough LPS and belonged to MLST ST 26. Interestingly, strain 12-05829 exhibited strong aggregative adherence but did not possess any known AAF genes^19^. This strain also did not contain the AggR regulon, which is important for the control of a number of genes involved in virulence encoded on pAA and chromosomal EAEC pathogenicity islands^19,20^. EAEC strains negative for *aggR* are commonly designated as atypical EAEC^21,22^.

The timely detection of Shiga-toxigenic hybrids is important because of their high pathogenicity^6^. Whereas recognition of *stx* and its variants is well established, the determination of adhesion or aggregation factors as observed in LEE-negative *E. coli* pathovars is still challenging due to their diversity^23,24^. Therefore, understanding such variants and their pathogenicity is essential for EHEC risk evaluation. In this study, we investigated the genetic basis of aggregative adhesion in the Shiga-toxigenic hybrid strain 12-05829 and assessed the consequences of its knockout. As a result, we identified genes for a novel type of fimbriae, designated aggregate-forming pili (AFP), located on a plasmid harboring marker genes for EAEC. We showed that the *afp* genes are responsible for bacterial piliation, autoaggregation, adhesion, and cytotoxicity and are present in a variety of intestinal pathogenic *E. coli* from human infections.

## Results

### Shiga-toxigenic hybrid strain 12-05829 carries EAEC marker genes but not AAF genes

Although the LEE-negative Shiga-toxigenic hybrid strain 12-05829 shows aggregative adherence to HEp2 cells, no genes encoding AAF/I–V were detected by a PCR-based analysis^19^. Therefore, we performed whole genome sequencing to identify the genes involved in aggregative adhesion. The major genomic characteristics of this strain are summarized in Table 1 and are shown in comparison to the 2011 EHEC/EAEC O104:H4 outbreak strain.

**Table 1:**
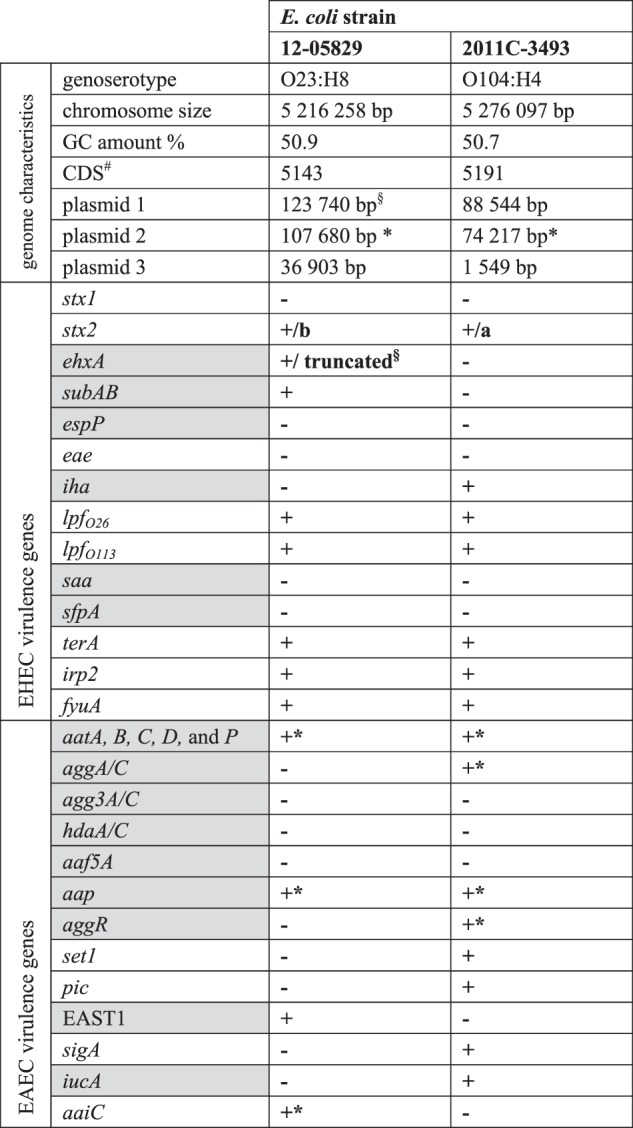
Major genome characteristics and virulence genes of the Shiga-toxigenic hybrid strain 12-05829 and the outbreak strain O104:H4 from 2011 (NCBI: NC_018658.1)

Gray shaded fields highlight genes typically encoded on plasmids

^#^CDS was determined by RAST annotation (http://rast.nmpdr.org/rast.cgi)

^§^*ehx* genes-containing plasmid

^*^aatA-containing plasmid

The genome sequence analysis showed that strain 12-05829 belongs to MLST ST26. We also performed genoserotyping by extracting the sequences of the *wzx* and *wzy* genes which belonged to serogroup O23. The *fliC* gene was assigned to serotype H8. Similar to O104:H4, O23:H8 is a very rare serotype, with only one case of human STEC infection with an O23 strain reported in the USA from 2004 until 2014 and only one described by the German NRC from 1997 until 2018^25^.

**Fig. 1:**
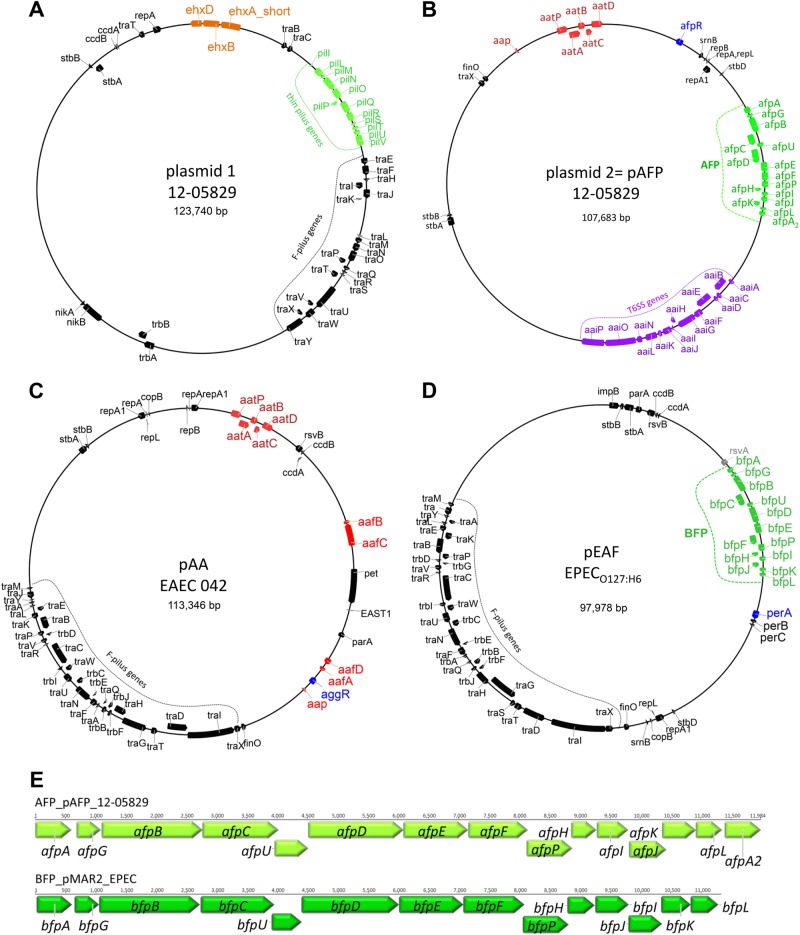
Shiga-toxigenic hybrid strain 12-05829 harbors an *aatA*-positive plasmid containing a novel fimbrial locus (*afp*). Plasmid 1 of strain 12-05829 (**a**) carries genes for the F-pilus, the thin pilus (shown in green) and an incomplete *ehx* operon (shown in orange) containing a truncated *ehxA*. Plasmid 2 of EHEC/EAEC strain 12-05829 (**b**) carries genes typical for EAEC strains (*aatA, B, C, D*, and *P; aap;* shown in red) which are also encoded on the pAA plasmid of strain Ec042 (GenBank: NC_017627.1) (**c**) and additionally contains an operon encoding an aggregate-forming pilus (*afp*) with limited similarity to the *bfp* operon of EPEC strains (shown in green) located on the pEAF plasmid (GenBank: NC_011603.1) (**d**). Plasmid 2 of strain 12-05829, pEAF and pAA all encode AraC-family regulator proteins (shown in blue). **e** Schematic overview of the *afp* operon structure of the Shiga-toxigenic hybrid strain 12-05829 compared to the *bfp* operon of the EPEC strain O127:H6 (80). The *afp* locus encodes the 14 classical constituents of a pilus locus (*afpA - afpL* and *afpU*) and contains an additional gene homologous to *afpA*, designated *afpA2*.

Shiga-toxigenic hybrid 12-05829 comprised an ~5.2 Mb chromosome and three plasmids (~124, ~108, and ~37 kb), with the 124 kb plasmid (plasmid 1) exhibiting some features of the hemolysin-coding plasmid of EHEC and the 108 kb plasmid (plasmid 2) harboring *aatA* (Table 1, Fig. 1a, b). Of note, *aatA*, which is part of the *aatA-D* operon located on the pAA plasmid (Fig. 1c), is commonly used as a diagnostic marker for EAEC^18^.

Several chromosomal- or plasmid-located EHEC and EAEC marker genes were identified in strain 12-05829 that were also present in the EHEC/EAEC O104:H4 outbreak strain (Table 1). Typical marker genes for EAEC, such as *aap* (on plasmid 2), and *east1* (chromosomal localization) were detected in strain 12-05829 in addition to the *aat* operon (Table 1, Fig. 1b). However, genes coding for the known types AAF (I–V), which are typically located on pAA, were not identified within the *aatA*-containing plasmid 2 or the chromosome. The structure of the *aatA*-harboring plasmid 2 of strain 12-05829 compared to pAA of EAEC 042 and pEAF of EPEC O127:H6 is shown in Fig. 1 (Fig. 1b–d), as is plasmid 1 of strain 12-05829, which contains parts of the enterohemolysin operon (Fig. 1a). Of note, plasmid 2 of strain 12-05829 was also observed to carry a putative type VI secretion gene operon (*aaiA-P*, except *aaiM*) (Fig. 1b). Typically, the *aai* operon is chromosomally localized in EAEC and is implicated in pathogenicity^26,27^. The *aaiC* gene encodes a secreted protein and is commonly used as chromosomal EAEC marker^27^.

### Plasmid 1 carries a *pil* operon that is not essential for aggregative adherence

We subsequently screened the chromosome and plasmid sequences for genes potentially contributing to aggregative adherence. We observed that plasmid 1 of the Shiga-toxigenic hybrid strain 12-05829 carries a type IV pilus biosynthesis locus (*pil*) consisting of 11 genes (*pilL* to *pilV* and *pilI*) (Fig. 1a). The *pil* locus has >99% nucleotide identity to plasmid-coded *pil* regions of other pathogenic *E. coli* deposited at NCBI, such as the O113:H2 98NK2 and EH41 STEC strains (GenBank: AF399919 and AY258503). However, deletion of the *pil* locus in plasmid 1 of strain 12-05829 did not affect bacterial autoaggregation or adhesion to HEp-2 cells (Fig. S1), similar to the results observed for the O113:H2 98NK2 strain after *pil* plasmid curing^28^.

### Plasmid 2 contains a novel *afp* operon encoding proteins with limited similarity to the bundle-forming pilus of EPEC as well as a novel AraC-like regulator gene

Interestingly, the *aatA*-containing plasmid 2 (Fig. 1b) was observed to carry genes encoding proteins with some homology to the bundle-forming pilus (BFP) apparatus of enteropathogenic *E. coli* (EPEC). Typical EPEC strains contain an EPEC adherence factor plasmid (pEAF) with a *bfp* operon coding for type IV pili (Fig. 1d)^29^. BFP are required for the formation of adherent microcolonies in a pattern known as localized adherence and for full EPEC virulence^29^. Fourteen genes (*bfpA, G, B, C, U, D, E, F, P, H, I, J, K*, and *L*) are arranged in an operon in pEAF, such as pMAR2 of EPEC O127:H6, as well as three genes coding for regulatory proteins (*bfpT, V*, and *W*; designated *perA, B*, and *C* in pMAR2) that are necessary for the formation of functional BFP^29^. A similarly organized operon consisting of 15 genes, designated here as AFP, was identified on plasmid 2 of EHEC/EAEC 12-05829. Therefore, plasmid 2 was named pAFP (Fig. 1b, e). Interestingly, two homologues of the EPEC *bfpA* gene are present in the *afp* operon at the first (designated *afpA*) and last position (designated *afpA2*) (Fig. 1e, Supplementary Table S1). The average nucleotide identity of this novel operon compared to *bfp* operons described by Nataro et al. in 1987 and Tobe et al. in 1999 was approximately 52% (36-59%)^30,31^ (Supplementary Tables S1 and S2), whereas the identity between classical *bfp* operon genes is typically >98% (Supplementary Table S2). The limited relatedness of the classical EPEC *bfp* genes to the novel *afp* genes of strain 12-05829 explains why it was previously not possible to detect the *afp* operon by means of routine PCR based on the *bfpA* sequence from EPEC strain B171^32^. In EPEC, *bfpA* encodes the major structural subunit of the bundle-forming pilus^29^. Although the protein relatedness was even lower (average 42%, 18–60%) than nucleotide identity, many AFP proteins share conserved domains with BFP proteins. This observation suggests that although AFP are clearly distinct from EPEC BFP, they may have similar functions (Supplementary Table S1).

The expression of the EPEC *bfp* operon is regulated by BfpT/V/W, which is also designated as PerA/B/C and is encoded on pEAF (Fig. 1d)^29^. BfpT (PerA) belongs to the AraC family of transcriptional activators and is required for the autoactivation of the *per* operon^29^. The *aatA*-containing plasmid 2 (pAFP) of strain 12-05829 was not observed to harbor an EAEC *per* operon or *aggR* but was shown to carry a gene encoding a protein with an AraC family regulatory helix-turn-helix domain (Fig. 1a). The novel putative AraC-regulator, designated as AfpR, is only 28% identical to BfpT (PerA) of the classical EPEC and 38% identical to AggR of EAEC. Thus, we identified a novel *aatA*-containing-plasmid-localized *afp* operon encoding proteins with limited similarity to the bundle-forming pilus of EPEC as well as a novel putative AraC-like regulator gene.

### Identification of novel pilus structures that promote a high degree of autoaggregation

As typical EPEC and EAEC strains are capable of autoaggregation^20,23,29,33^, we wanted to assess the degree and pattern of autoaggregation for strains containing the *afp* operon. Specifically, we compared the Shiga-toxigenic hybrid strain 12-05828 and two additional *afp*-positive *E. coli* strains harboring *aatA* but not the AAF genes from the NRC collection (strains 12-05898 and 14-01010) to typical EPEC, EAEC strains with AAF I–V, and a negative control *E. coli* K12 C600 strain (Table 2).

**Table 2:** Selected *E. coli* strains used in this study

	Strain designation	Serovar	Pathovar	Material	Clinical symptoms	WGS type	MLST ST	*afp* operon^a^	autoaggregation	*stx*	*eaeA*	*ehxA*	*subAB*	*aatA*	References
EHEC/EAEC *afp*-positive^a^	12-05829	Orough:H-geno-serotype: O23:H8	EHEC/ EAEC	human, stool	diarrhea	PacBio/ MiSeq	26	+	+	+ 2b	−	+ truncated	+	+	^19^ and this study
Further *afp*-positive^a^	12-05898	O118:Hnt	EAEC	human, stool	diarrhea	MiSeq	10	+	+	−	−	−	−	+	this study
	14-01010	O66:H25	EAEC	human, stool	n.s.	n.d.	10	+	+	−	−	−	n.d.	+	this study
*bfp*-positive	01-05814	O127:H6	EPEC	human, stool	diarrhea	n.d.	15	−	+	−	+	−	n.d.	−	^59^
AAF I	11-02027, Outbreak 2011	O104:H4	EHEC/ EAEC	human, stool	HUS	n.d.	678	−	+	+ 2a	−	−	−	+	^2^
AAF II	*E. coli* 042	O44:H18	EAEC	human, stool	diarrhea	n.d.	414	−	+	−	−	−	−	+	^49^
AAF III	01-09591	O104:H4	EHEC/ EAEC	human, stool	HUS	n.d.	678	−	+	+ 2a	−	−	n.d.	+	^11^
AAF IV	10-06235	O59:H-	EHEC/EAEC	human, stool	bloody diarrhea	n.d.	1136	−	+	+ 2a	−	−	−	+	^19^
AAF V	10-03550	O111:H21	EAEC	human, stool	n.s.	n.d.	40	−	+	−	−	−	n.d.	+	^19^
negative control	K12 C600	O16:H48	non pathogenic	n.s.	n.s.	n.d.	10	−	−	−	−	−	−	−	^60^

For further strains refer to Supplementary Table S5 and S7

*n.s.* not specified, *n.d.* not determined, + positive, − negative

^a^tested for *afpA2, B, D* and *P* genes (*afp* operon markers) by PCR

**Fig. 2:**
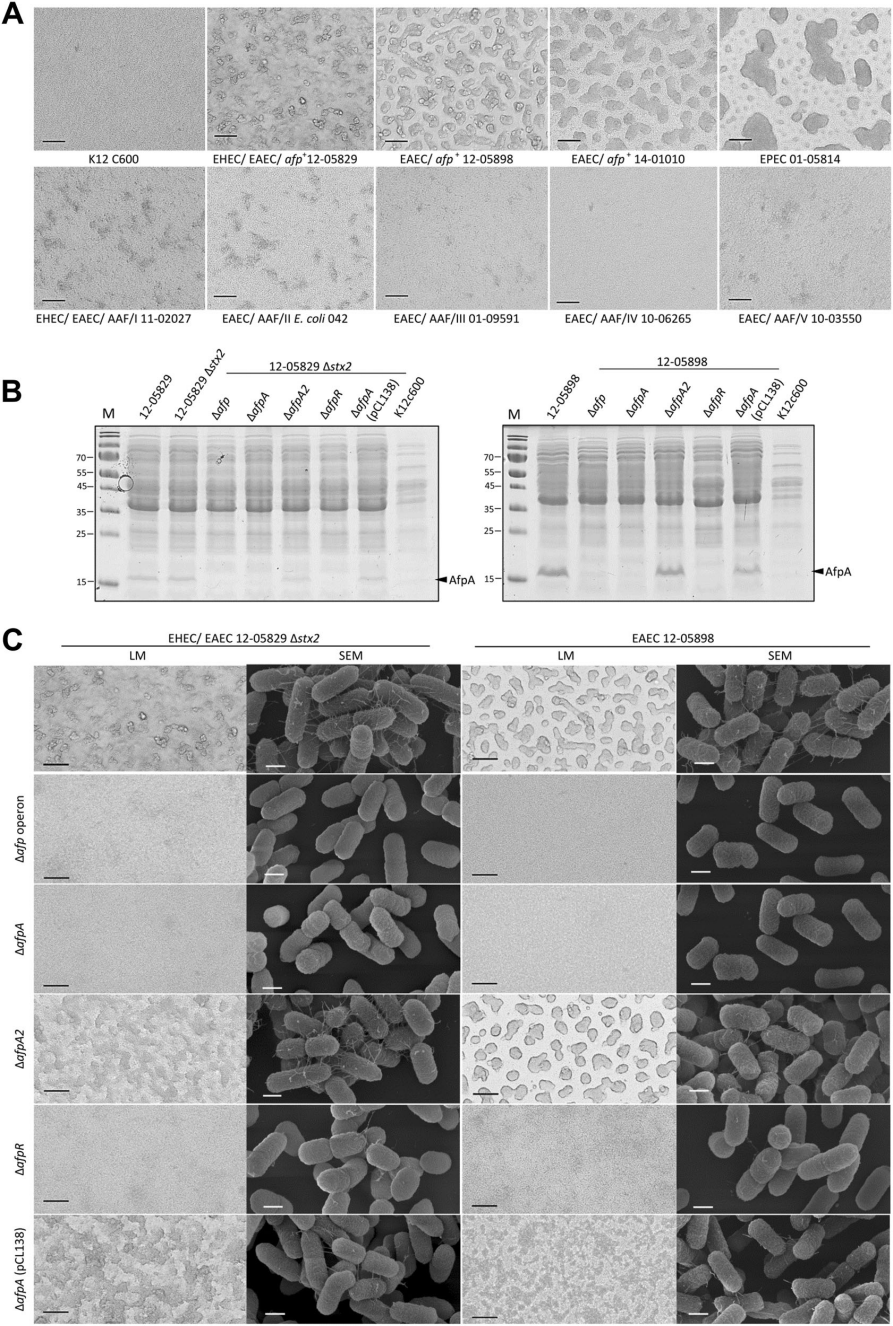
*afp*-positive *E. coli* strains exhibit a high degree of autoaggregation. Deletion of *afp*, *afpA* and *afpR* results in loss of AfpA abundance, autoaggregation and pilus formation. **a** Light microscopy images of autoaggregating *E. coli* K12 C600, the *afp*-positive strains 12-05829, 12-05898, 14-01010, EPEC 01-05814 and AAF/I–V-positive strains. Formation of large bacterial aggregates is evident for the *afp*-positive strains 12-05829, 12-05898 and 14-01010 as well as for the EPEC strain 01-05814. **b** Cell lysate proteins of *afp*-positive *E. coli* strains 12-05829 and 12-05898, their respective *afp*, *afpA*, *afpA2*, and *afpR* deletion mutants and the Δ*afpA* complementation strain carrying pCL138 separated by SDS-PAGE. **c** Autoaggregation of the *afp*-positive *E. coli* strains 12-05898, 12-05829 Δ*stx2*, the respective *afp, afpA*, *afpA2* and *afpR* deletion mutants and the *ΔafpA* complementation strain carrying pCL138. Light microscopy (LM) images for (**a**) and (**c**) were captured with 200-fold magnification (scale bar 100 µm) and scanning electron microscopy images for (**c**) with 50,000-fold magnification (scale bar 500 nm). M molecular weight marker in kDa

We observed a strong degree of autoaggregation for all *afp*-positive strains after 3 h of incubation but not for the K12 C600 strain. The autoaggregation phenotype of the *afp*-positive strains (12-05829, 12-05898, and 14-01010) did not resemble that of the AAF I–V-positive EAEC, which produced smaller and more diffuse aggregates, but the phenotype was similar to that of the EPEC strain producing more prominent aggregates (Fig. 2a). Although this result suggested a functional similarity between AFP and BFP, which was also suggested by their shared protein domains, a clear phenotypic difference was observed between AFP and AAF. Furthermore, we constructed mutants in two *afp*-positive strains (Shiga-toxigenic hybrid 12-05829 Δ*stx2* and EAEC 12-05898) in which the entire *afp* operon, *afpA*, *afpA2*, or *afpR* were deleted. To verify the mutants, the presence of the major pilin AfpA was assessed by SDS-PAGE. We detected the characteristic ~18 kDa protein in all samples except in the ∆*afp* operon, ∆*afpA*, and ∆*afpR* mutants, and complementation was achieved by the introduction of the pCL138 plasmid into the ∆*afpA* mutant (Fig. 2b). Accordingly, we observed a lack of autoaggregation in the ∆*afp* operon, ∆*afpA*, and ∆*afpR* strains but not in the ∆*afpA2* knockout mutant (Fig. 2c). Scanning electron microscopy (SEM) observations revealed the presence of fimbriae-like structures on highly aggregating bacteria of both the *afp*-positive unmodified bacterial strains and the ∆*afpA2* mutant, but they were absent in the ∆*afp* operon and ∆*afpA* mutants and exhibited a reduced occurrence in the ∆*afpR* mutant (Fig. 2c, more detailed presentation of pilus in Fig. S2). Reintroduction of the *afp* operon on plasmid pCL138 led to a restoration of aggregates and fimbriae in the ∆*afpA* mutants of both strain types (Fig. 2c). In summary, we showed that *afp*-positive strains displayed a high degree of autoaggregation that depended on the presence of a functional *afp* operon, the major pilin-encoding gene *afpA* and the regulatory protein-encoding gene *afpR*, but not *afpA2*.

### *Afp*-positive *E. coli* strains exhibit aggregative adherence to epithelial cells that is dependent on the *afp* operon, *afpA*, and *afpR* but not *afpA2*

**Fig. 3:**
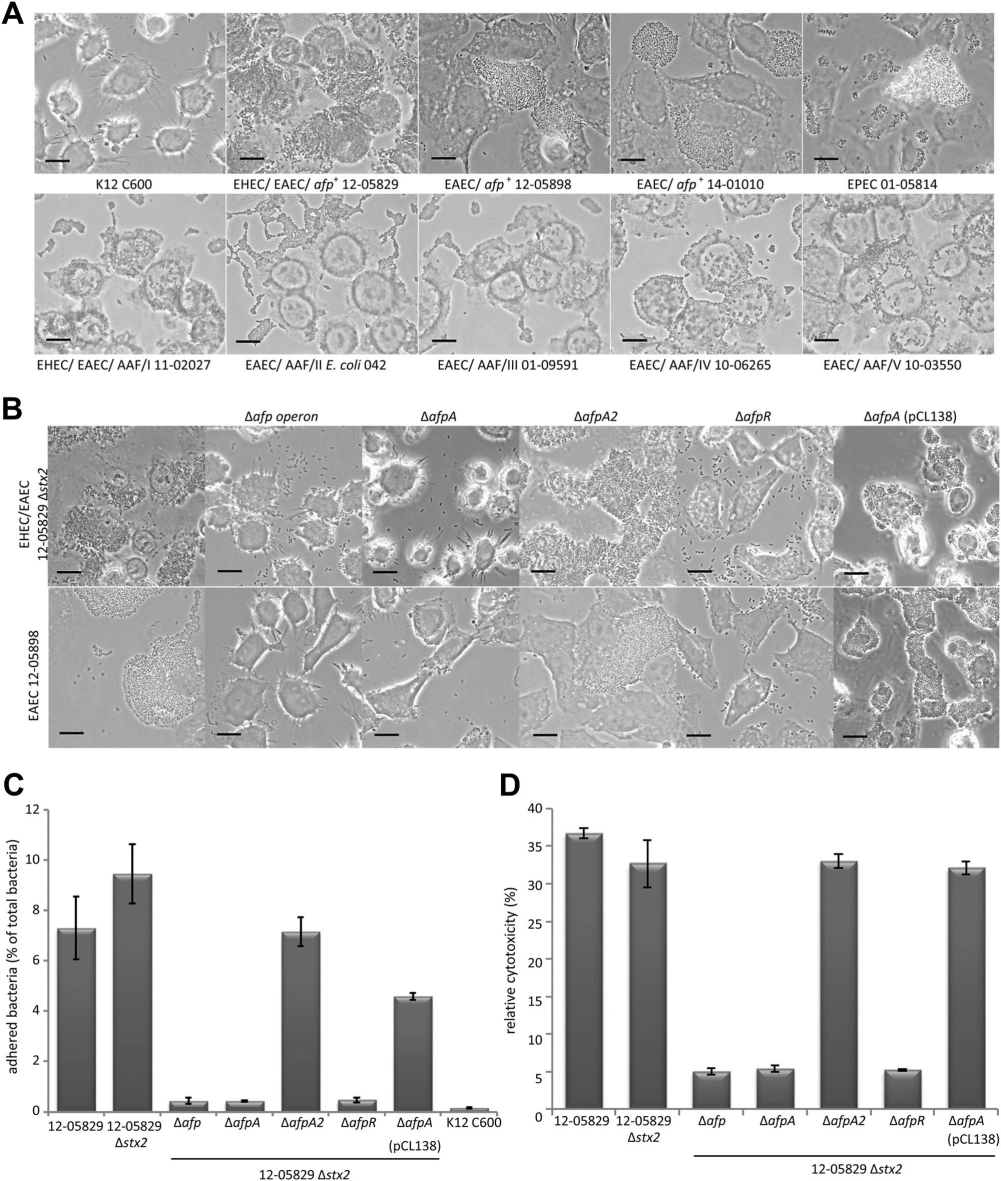
*afp*-positive *E. coli* strains exhibit a characteristic adherence pattern to HEp-2 cells and cytotoxicity depending on the presence of the *afp* operon and the AraC family regulator gene *afpR* but not *afpA2*. **a** Aggregative adherence to HEp-2 epithelial cells of the *afp-*positive *E. coli* strains 12-05829, 12-05898 and 14-01010 compared to EPEC 01-05814 and AAF/I–V-positive strains. Strain K12 C600, which lacks aggregative adherence, served as negative control. **b** Aggregative adherence to HEp-2 epithelial cells of the *afp*-positive *E. coli* strains 12-05898, 12-05829 Δ*stx2*, and the respective *afp*, *afpA*, *afpA2 and afpR* deletion mutants and the Δ*afpA* complementation strain carrying pCL138. Phase contrast microscopy images are shown with 1000-fold magnification (scale bar 25 µm) for (**a**–**c**) Quantitative HEp-2 adhesion assay of the Shiga-toxigenic hybrid strain 12-05829, the respective *stx2, afp*, *afpA*, *afpA2*, *afpR* deletion mutants and the Δ*afpA* complementation strain carrying pCL138. Bars show the percent of bacteria adhered to HEp-2 cells after 4 h. **d** Cytotoxicity assay performed with EHEC/EAEC strain 12-05829, its respective *stx2, afp*, *afpA*, *afpA2, afpR* deletion-mutants and the Δ*afpA* complementation strain carrying pCL138. The results for (**c**) and (**d**) represent the means and standard deviations of triplicate reactions and are representative for at least two additional experiments

We analyzed the pattern and degree of adherence of *afp*-positive *E. coli* to HEp-2 cells compared to that of AAF/I–V-positive EAEC, a BFP-positive EPEC strain, and a negative control *E. coli* K12 C600 strain. Whereas the K12 C600 strain did not show any adherence, the *afp*-positive strains exhibited a high degree of adherence (Fig. 3a). The adherence pattern of the *afp*-positive strains was more similar to that of the EPEC strain than to AAF/I–V-positive EAEC isolates. However, it is important to note that the aggregates of the *afp*-positive strains (12-05829, 12-05898, and 14-01010) on HEp-2 cells were unusually large and did not completely resemble those observed for EPEC, indicating that AFP indeed represent a distinct type of pili (Fig. 3a).

Subsequently, we tested the abovementioned mutants of the Shiga-toxigenic hybrid 12-05829 Δ*stx2* and EAEC 12-05898 strains for adherence to HEp-2 cells. We observed a lack of adhesion by the strains lacking ∆*afpA* and the ∆*afp* operon but not for the ∆*afpA2* knockout mutant (Fig. 3b). In addition, the deletion of *afpR* led to decreased adhesion. The reintroduction of the *afp* operon on a plasmid into the ∆*afpA* mutants restored the adhesion pattern for both strains (Fig. 3b). To quantify the extent of the adhesion, we performed an assay in which infected HEp-2 cells were washed and the amount of remaining bacteria was determined after 3 h of incubation (Fig. 3c). This assay clearly demonstrated that the lack of *afpA*, the *afp* operon, or  *afpR* but not *stx2* or *afpA2* led to dramatically reduced adherence of bacteria to HEp-2 cells (Fig. 3c). The *E. coli* K12 C600 strain did not show adhesion, and complementation of the ∆*afpA* mutant using pCL138 reestablished adhesion (Fig. 3c). In summary, we showed that *afp*-positive strains exhibited aggregative adhesion to HEp-2 cells and formed unusually large aggregates that depended on the *afp* operon, the major pilin gene *afpA*, and *afpR* but not *afpA2*.

### AFP are essential for cytotoxicity toward epithelial cells

In addition to being important for adhesion to host cells, pili may also cause cytotoxicity^34,35^. To assess the role of AFP in cytotoxicity, HEp-2 cells were incubated for 24 h with the Shiga-toxigenic hybrid strain 12-05829 and its mutants. The relative cytotoxicity was quantified via the detection of free lactate dehydrogenase from leaky HEp-2 cells. The results demonstrated the cytotoxicity of the wild-type strain, its *stx2*, and *afpA2* negative mutants and the *afp* complemented ∆*afpA* mutant, whereas the ∆*afpA*, ∆*afp* operon, and ∆*afpR* mutants were clearly less cytotoxic toward HEp-2 cells (Fig. 3d). These results demonstrate that AFP are essential for mediating cytotoxicity toward HEp-2 cells.

### Transformation of pAFP_12-05829_ into *afp*-negative *E. coli* K12 leads to bacterial autoaggregation and aggregative adherence toward epithelial cells

**Fig. 4:**
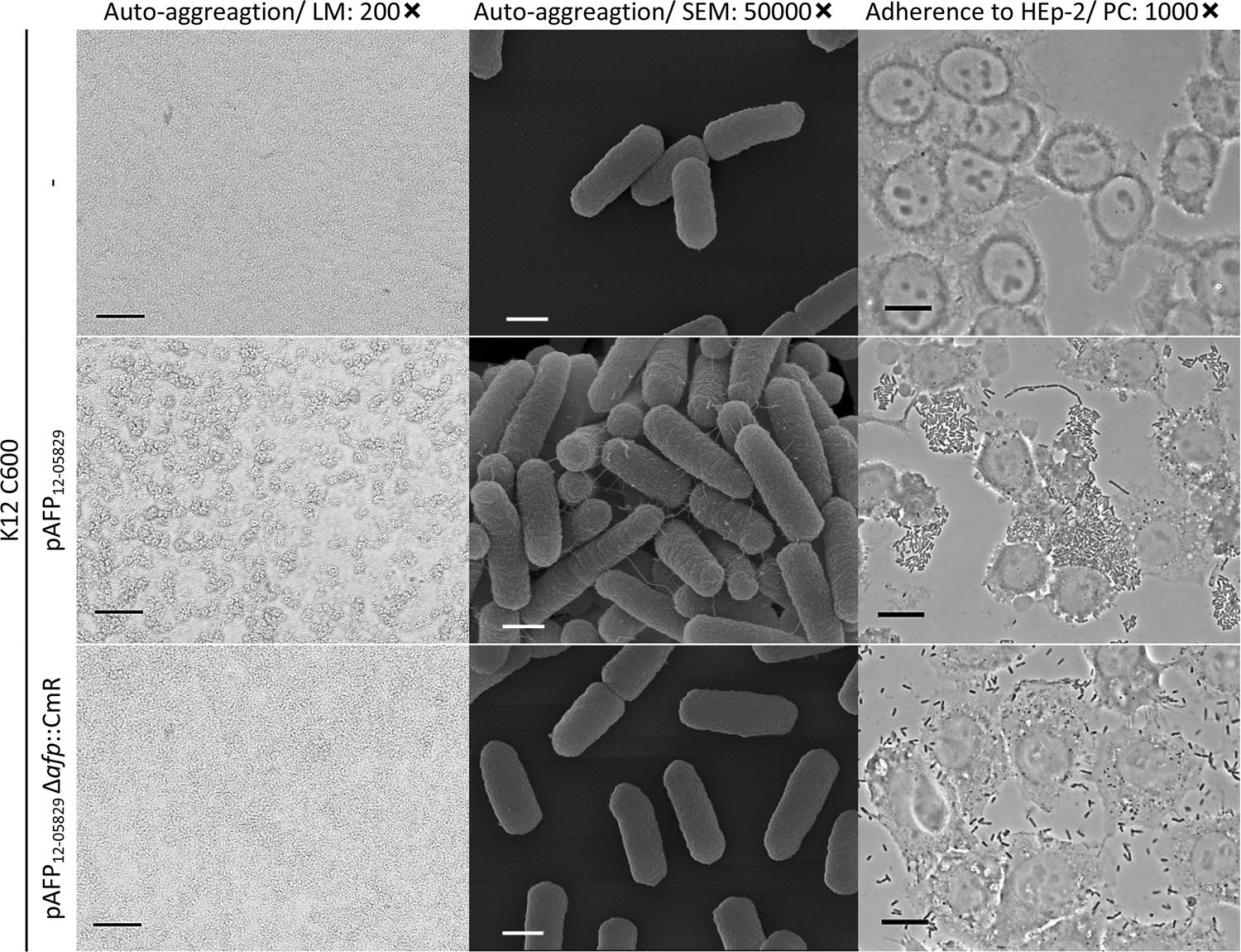
Nonpathogenic *E. coli* K12 C600 carrying pAFP_12-05829_ exhibits autoaggregation, pili structures, and adhesion to HEp-2 cells. The autoaggregation and aggregative adherence to HEp-2 epithelial cells are shown for K12 C600, K12 C600 (pAFP_12-05829_) and its respective *afp* deletion plasmid. Light microscopy (LM) images with 200-fold magnification (scale bar 100 µm), scanning electron microscopy (SEM) images with 50,000-fold magnification (scale bar 500 nm) and phase contrast microscopy images (PC) with 1000-fold magnification (scale bar 25 µm) are shown

Next, we tested whether the plasmid containing the *afp* operon and its associated phenotypes, such as autoaggregation and HEp-2 cell adhesion, could be transferred to an *afp*-negative *E. coli* strain. Thus, the plasmid pAFP_12-05829_ was labeled with a Cm^R^ cassette and isolated from the host strain for transformation into the *afp*-negative *E. coli* K12 C600 strain. Indeed, after the introduction of pAFP_12-05829_, the K12 C600 strain exhibited characteristic autoaggregation, fimbriae, and aggregative adhesion to HEp-2 cells (Fig. 4), whereas the deletion of the *afp* operon caused loss of these phenotypes (Fig. 4). This demonstrated that pAFP and the *afp* operon it harbors is sufficient to mediate autoaggregation and host cell adherence in other *E. coli* strains.

### *afp* operons are present in a variety of *aatA*-positive *E. coli* strains from human infections

We next assessed how widely distributed the *afp* operon is in the genomes of other *E. coli* strains. Using an NCBI nucleotide BLAST analysis with the strain 12-05829 *afp* operon as a query sequence, 17 *afp*-positive *E. coli* strains were identified (Supplementary Table S3) from all available *E. coli*, *Shigella* and *Citrobacter* genomes. Compared with strain 12-05829, the nucleotide identity of these *afp* operons compared was approximately 95.6–98.6%, confirming their close relatedness (Supplementary Table S4). All *afp*-positive strains were additionally positive for the *aat* operon, *aap*, and the gene encoding the new type of AraC family regulator (*afpR*), but these strains tested negative for *stx, eaeA, hlyA, aggR*, and *bfp* (Supplementary Table S3). Furthermore, in the NRC strain collection, we identified 35 *aatA*-positive but AAF/I–V gene- and *stx*-negative *E. coli* strains isolated from human infections between 2001 and 2014 (Table 2 and Supplementary Table S5). To our surprise, 26 of the strains were positive for the *afp* operon when analyzed for *afpB, D, P*, *A2* and *R* by PCR (Table 2 and Supplementary Table S5), indicating that *E. coli* strains harboring *afp* are regularly associated with human disease. Interestingly, these strains belong to a broad variety of serotypes and MLST STs (Table 2 and Supplementary Table S5). The genome of one of these strains (12-05898), which was used in this study for phenotypic assays (Figs. 2 and 3), was sequenced for a more detailed analysis of the *aatA*-containing pAFP. The MiSeq reads obtained for this strain were mapped to the pAFP_12-05829_ sequence and covered 90.7% of the reference with a pairwise identity of 95.5%. Importantly, the entire *aat* operon (nucleotide identity of 98.56%), *aap*, *afpR* and the *aai* operon genes were present in the sequence data of strain 12-05898.

**Fig. 5:**
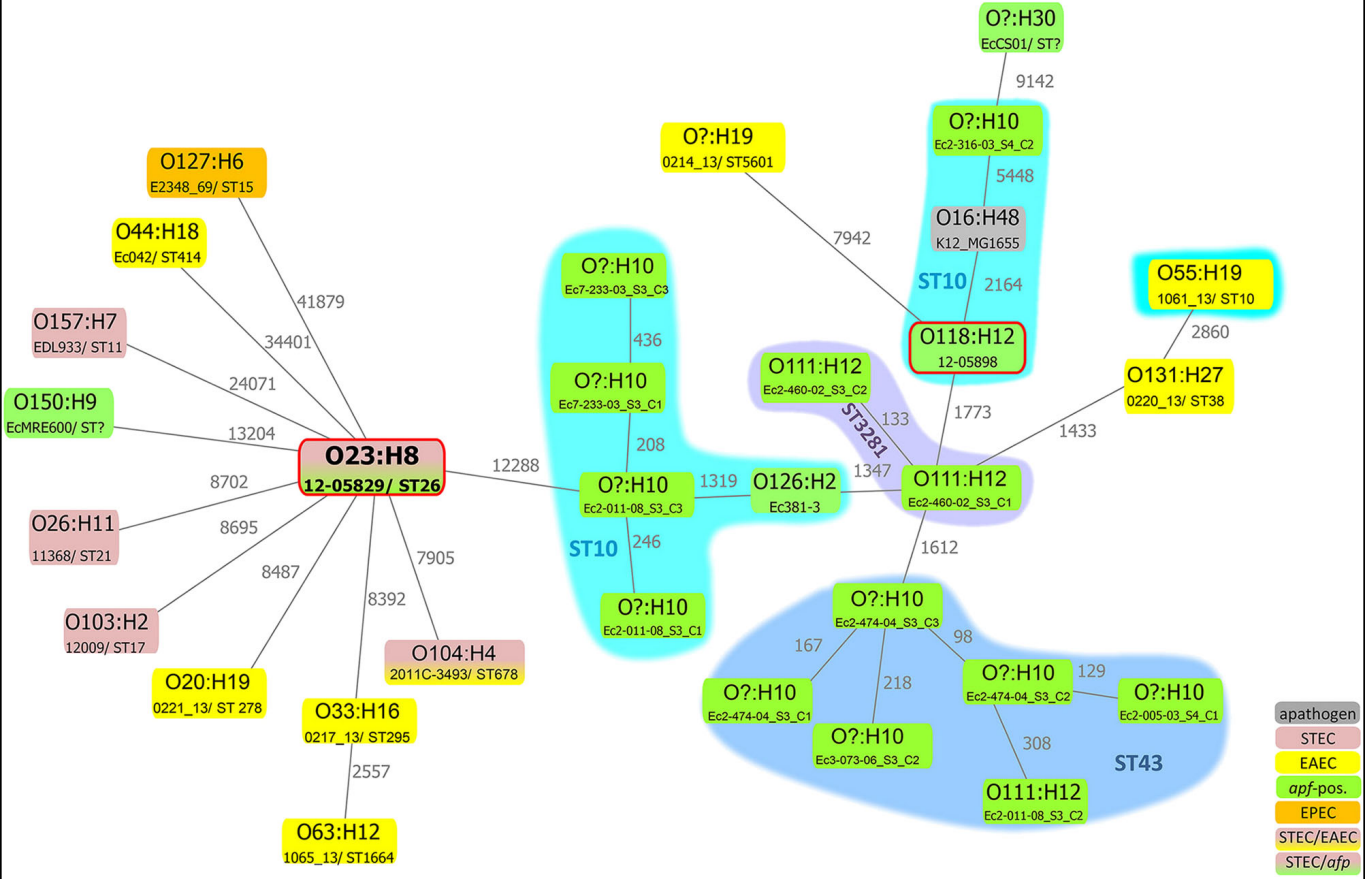
The Shiga-toxigenic hybrid strain 12-05829 and the EAEC strain 12-05898 do not exhibit a close phylogenetic relationship to other *afp-*positive strains from NCBI. Chromosomal phylogeny of *afp*-positive *E. coli* strains and examples of EAEC, STEC, EPEC and EHEC/EAEC strains in relation to the *afp*-positive EHEC/EAEC strain 12-05829 are shown, represented as a minimum spanning tree based on 94 553 core SNPs of 31 strains. The different *E. coli* strains are phylogenetically diverse or cluster due to their sero- or MLST sequence-type, but not due to their pathovar. All sequences, except those of strain 12-05829 and 12-05898 (red frame), were obtained from NCBI (Supplementary Table S3). The gray numbers next to the lines represent the SNP count. ST? = unassigned MLST ST. O? unassigned serogroup

The chromosome sequence of the Shiga-toxigenic hybrid strain 12-05829 was phylogenetically compared to selected EHEC/STEC (O26:H1, O103:H2, and O157:H7), EAEC (O44:H18 strain 042 and recently published EAEC genomes^36^), STEC/EAEC (O104:H4), EPEC (O127:H6) and other *afp*-positive *E. coli* strains (Table 2 and Supplementary Table S4), as well as the nonpathogenic *E. coli* K12 MG1655 (O16:H48) strain using SNP-based analysis (Fig. 5). Although still considerably distant, the most closely related *E. coli* genome to strain 12-05829 among these strains was that of EHEC/EAEC O104:H4 from 2011. SNP analysis further revealed that the *E. coli* strains did not cluster due to their pathovar or the presence of *afp* genes (Fig. 5). In summary, the novel *afp* operon is present in a variety of other *aatA*-positive *E. coli* strains, and the Shiga-toxigenic hybrid strain 12-05829 is only distantly related to other strains harboring the *afp* operon.

## Discussion

**Fig. 6:**
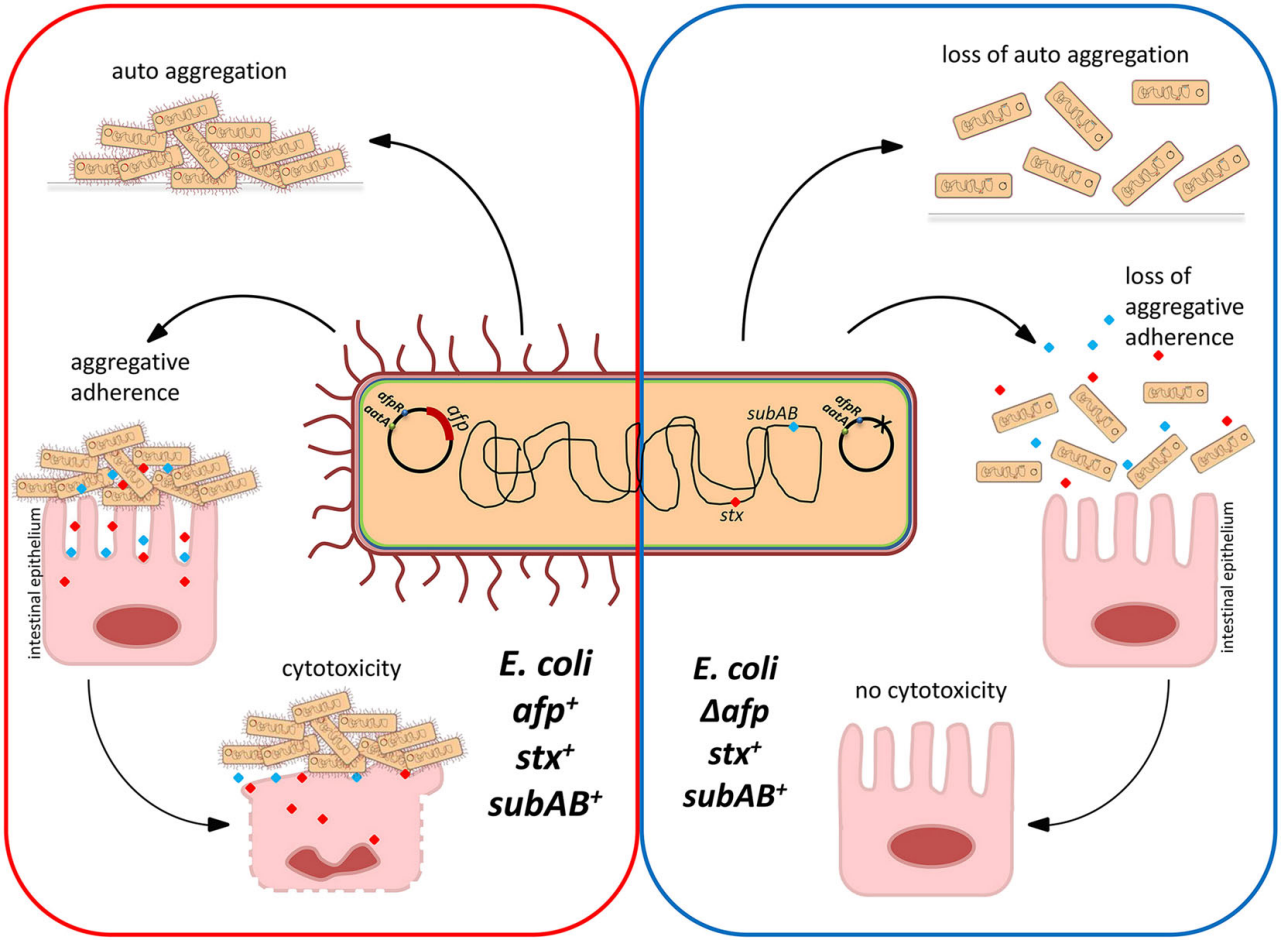
Summarized characteristics of an *afp*- and cytotoxin gene (*stx2* and *subAB*)-expressing LEE-negative *E. coli* (red frame) compared to an *afp* deletion mutant (blue frame). Red and blue dots represent potential toxins, such as SubAB or Shiga toxin

In this study, a detailed genomic analysis of the Shiga-toxigenic hybrid strain 12-05829 resulted in the identification of an *aatA*-containing plasmid, pAFP, encoding the novel pili locus *afp*, which is essential for bacterial aggregation, aggregative adherence to host cells, and mediation of cytotoxicity (Fig. 6). Interestingly, *afp*-encoded proteins showed a high degree of identity (average of ~99%) among different *afp*-positive strains but a lower identity (average of ~42%) to EPEC BFP proteins (Supplementary Table S2), indicating that the novel AFP are only distantly related to BFP.

The *afp* locus encodes the 14 classical constituents of a pilus operon (*afpA* to *afpL* and *afpU*) as well as an additional homologue of the major pilin gene *afpA*, *afpA2* (Fig. 1e)^29^. Although AfpA and AfpA2 share ~60% protein identity, AfpA was the only abundant major pilin detected in this study that contributed to AFP pilus generation and the consequent autoaggregation, adhesion, and cytotoxicity phenotypes. Therefore, the role of AfpA2 is still unclear and remains a subject for further research.

AFP pili exhibited considerable cytotoxicity toward HEp-2 cells (Fig. 3d). On the one hand, pili promote clustering of bacterial cells into large aggregates that nearly cover the entire host cell, allowing bacteria to secrete toxins at high localized concentrations. In addition, the clusters further hamper the diffusion of toxins to the cell exterior. On the other hand, pili assure close proximity between bacteria and the host cell, which may allow the efficient application of secreted toxins and other factors directed toward the host cell (Fig. 6). Similar effects to those that were observed for AFP have been described for EPEC. An EAF-negative EPEC strain not expressing BFP was observed to be less cytotoxic than EAF-positive EPEC^35^. Pili-mediated cytotoxicity is not limited to intestinal pathogenic *E. coli*. Recently, Basso et al. showed that pili promote close contact between bacteria and eukaryotic cells and the localized action of the exolysin ExlA during infection with *Pseudomonas aeruginosa*. Such an intimate interaction is required for the formation of pores and subsequent eukaryotic cell death^37^, a scenario that may be of similar importance for the Shiga-toxigenic hybrid described in this study.

In our experiments, no major difference in cytotoxicity between the *afp*-positive Shiga-toxigenic strain 12-05829 and its *stx2* knockout mutant was noted (Fig. 3d). This observation may be explained by the far lower toxicity of Stx2b of strain 12-05829 compared to Stx2a^38^, which is caused by the weak binding of Stx2b to the Shiga toxin receptor globotriaosylceramide Gb(3)^39^ and its limited capability for proteolytic activation by furin^38^. These observations raise the question of what other toxin may be responsible for the observed cytotoxicity? The Shiga-toxigenic hybrid strain 12-05829 possesses *subAB* (Table 1), encoding the subtilase AB5 cytotoxin produced by some STEC, especially LEE-deficient strains^40^. Because SubAB is known to induce apoptosis and cell death in HeLa cells^41^, we hypothesize that SubAB has a toxic effect on HEp-2 cells triggered by AFP. Another factor that may be involved in the observed cytotoxicity is EAST1 (EAEC heat stable enterotoxin), encoded by *astA*, which activates membrane-bound guanylate cyclase and causes downstream effects on several signaling pathways, ultimately leading to loss of electrolytes and water in intestinal cells^42^.

We also identified and characterized a gene encoding a putative regulator of the *afp* operon in pAFP, designated *afpR*, which similar to AggR from EAEC and BfpT from EPEC contains an AraC-regulator family conserved domain (Fig. 1b)^43^. Indeed, pilus structures and pilin protein, as well as the associated autoaggregation, adhesion, and cytotoxicity were reduced in the ∆*afpR* mutants of *afp*-positive *E. coli* strains. The effects of the *afpR* gene deletion confirm that the novel AraC regulator family protein is important for the formation of pili and likely plays a similar role as BfpT (PerA) in EPEC and AggR in EAEC^44,45^. The presence of a gene that is homologous to *afpR* on a plasmid with *aatA* and *aap* may therefore be of relevance in atypical EAEC^21,22^. Atypical EAEC strains are characterized by a variety of EAEC marker genes, such as *aatA* and *aaiC* but are negative for *aggR*. It is therefore conceivable that another AraC family regulator, such as the one identified in this study, may regulate gene expression. This possibility can now be easily tested using the PCR method described in this study (Supplementary Table S6). Atypical EAEC are frequently identified, and the *aatA*/*afp*-positive pathogenic *E. coli* described here likely belong to this category. For example, in a Brazilian study of the etiology of acute diarrhea in children, 8% of the *aatA*-positive EAEC strains were *aggR*-negative^26^. In a study by Lima et al. 2013, the classical *aggR* gene was only observed in 56.7% of the EAEC-positive samples from Brazil^27^, raising the question of whether the residual >40% of samples contained *afpR*. Further investigations of the AfpR regulator are currently in progress to examine whether it acts similar to AggR, which activates the expression of many genes in EAEC strain 042, including chromosomal and plasmid-borne loci^44,46,47^.

The *afp* locus was also identified in 17 strains deposited at NCBI and in 26 *aatA*-positive and AAF/I–V gene-negative *E. coli* from the German NRC strain collection. Of these 43 strains, all were *aatA*-positive, suggesting that the *afp* operon is located on a similar pAFP plasmid (Table 2, Supplementary Tables S3 and S5). Indeed, an alike pAFP was detected in the EAEC strain 12-05898 investigated in this study, because the complete *afp* operon was present and the MiSeq reads that covered more than 90% of the pAFP_12-05829_ sequence. This result is in accordance to the observation that *afp*-associated properties may be transmitted by means of plasmid transfer to other *E. coli* (Fig. 4). For the remaining 25 *afp*- and *aatA*-positive strains from the NRC collection we confirmed *afp* operon plasmid localization, because the presence of *afpB, afpD, afpP* and *afpA2* were detected by PCR with isolated plasmid DNA. A phylogenetic comparison of the *afp*-positive strains did not reveal a close relationship (Fig. 5). Therefore, it is conceivable that the *afp*-containing plasmid is taken up by *E. coli* strains, such as by conjugative plasmid transfer. In summary, our findings demonstrate that the novel *afp* locus is widely distributed among *aatA-*positive and AAF/I–V gene-negative *E. coli* strains and is present in a novel plasmid among *aatA*-containing plasmids that are unrelated to the *aatA*- and AAF-containing plasmid pAA of EAEC and the *aatA*-negative *bfp*-containing plasmid pEAF of EPEC^48,49^.

None of the *aatA*- and *afp-*positive strains from NCBI or the NRC collection were *stx*-positive, indicating that the Shiga-toxigenic hybrid strain 12-05829, such as the outbreak strain from 2011, represents the rare occurrence of hybrid strains combining *stx2* with an extraordinarily strong aggregative adherence. The clinical symptoms of the patient carrying strain 12-05829 at the time of sampling were diarrhea without the excretion of blood or HUS development. Strain 12-05829 carries the *stx2b* gene, and the *stx2b*-derived toxin is known to be at least 25-times less potent than those derived from *stx2a* and *stx2d*^38^. Nevertheless, *stx2b*-containing strains have been isolated from HUS patients at NRC in two cases, one strain also represented in the HUSEC collection (HUSEC028) harboring *stx2b* in combination with *stx1c* and another previously reported strain harboring *stx2b* in combination with *stx1a*^11,38,50,51^. However, since the *afp* operon-carrying strains appear diverse, other types of *stx* with more severe pathogenic potential may also be acquired by means of bacteriophage incorporation. In contrast, STEC strains may also take up pAFP to generate STEC/AFP hybrids, which may have a higher virulence potential.

In summary, in this study we showed that AFP are important and widely distributed adhesion and virulence factors that should be included into the adhesion factor panel used to analyze LEE-negative STEC and for risk evaluation. As the AFP genes studied here are located on a novel *aatA*-containing plasmid, horizontal gene transfer of the AFP operon to other *E. coli* strains and even other species is conceivable and may be important for aggregative adherence phenotypes, as shown for the *E. coli* K12 C600 strain (Fig. 4) and to increase toxic effects.

## Materials and Methods

### Strains used in the study

The strains used in the study are listed in Supplementary Table 2, Supplementary Tables S5 and S7. The strains outlined in Table 2 and Supplementary Table S5 were, in most cases, collected by the German National Reference Centre (NRC) for *Salmonella* and other Bacterial Enteric Pathogens and were routinely analyzed for the phenotypic serovar, *stx* gene type, *eaeA*, AAF type and MLST ST as described previously^19^. Unless otherwise stated, strains were grown on nutrient agar (Oxoid GmbH, Germany) or Luria Bertani (LB) broth or agar.

### Whole-genome sequencing (WGS)

Whole-genome sequencing of Shiga-toxigenic hybrid strain 12-05829 was performed by GATC Biotech (Konstanz, Germany) using a PacBio RS II sequencer (Pacific Biosciences, USA), which produces long read sequences. DNA was isolated by using a Qiagen Genomic-Tip 100/G Kit (Qiagen, Germany) according to manufacturer’s instructions, and 10 µg was sent to GATC. Additionally, short read genome sequencing was performed using an Illumina MiSeq benchtop sequencer in paired-end mode with a v3 chemistry-based cartridge 600 (600-Cycle Reagent Kit, Illumina, Germany). In this case, DNA from the *E. coli* strains EHEC/EAEC 12-05829 and EAEC 12-05898 was isolated with a Qiagen DNeasy Blood & Tissue Kit (Qiagen, Germany) according to manufacturer’s instructions, and 1 ng of the extracted DNA was used to generate libraries using the Nextera XT DNA Library Preparation Kit from Illumina (FC-131-1024). The sequences were uploaded to the European Nucleotide Archive (ENA) in study Acc. No. PRJEB28343 (Acc. no. ERS2673049 for MiSeq reads of strain 12-05829; Acc.no. ERS2673050 for MiSeq reads of strain 12-05898; and Acc.no. ERS2673049 for PacBio contigs of strain 12-05829).

### Bioinformatics analyses

De novo assembly of the PacBio sequence data was performed by GATC utilizing HGAP3 (Pacific Biosciences). The polished assembly yielded 3 plasmid contigs and 5 contigs belonging to the chromosome with a 41- to 63-fold coverage. Based on the overlapping ends of the 3 plasmid contigs, it was possible to close the plasmid sequences, which was verified by PCR (for primers see Supplementary Table S6).

EHEC, EAEC, and enteropathogenic *Escherichia coli* (EPEC) marker genes (Table 1) were searched for by mapping the trimmed Illumina reads against the respective gene sequences downloaded from NCBI with Geneious R10.0.5 (Biomatters Limited, Auckland, New Zealand) (map to reference function), where a coverage of 100% of the reference sequence and 95% sequence homology was set as the threshold. Second, we checked for homologs of the AAF/I to AAF/V and BFP genes using Geneious R10.0.5 (annotate function) with a cut-off of 80% identity. Third, we translated the open reading frames of the Shiga-toxigenic hybrid strain 12-05829 plasmids 1 and 2 into protein sequences and searched for homologs using NCBI pBLAST (standard settings). Using the third strategy, the *pil* genes were identified on plasmid 1, and the novel *afp* genes with limited homology to *bfp* were identified on plasmid 2. An in-house pipeline was used for phylogenetic analysis, including (I) read trimming using Trimmomatic (vers. 0.32) with default parameters, (II) alignment of trimmed reads to reference sequence using BWA mem with default parameters (version 0.7.10-r789), (III) sam file to bam file conversion using samtools (version 0.7.10-r789), (IV) pileup using samtools mpileup (without probabilistic realignment for the computation of base alignment quality), (V) variant calling using VarScan (vers. 2.3: parameters: min-coverage, 10; min-reads, 8; min-avg-qual, 20; min-var-freq, 0.8; min_freq-for-hom, 0.8; p-value, 0.01; and strand-filter disabled), and (VI) consensus sequence creation. To build the consensus sequences, insertions were excluded, and base calls were only considered if supported by at least 80% of the reads (otherwise N was called). SNPs were filtered using a previously published SNP filter^52^.

The pseudosequences of polymorphic positions were used to create a minimum spanning tree with Phyloviz^53^. To include complete genome sequences from NCBI to phylogenetic analysis, the sequences were converted to artificial FASTQ reads using artfastqgenerator^54^ and were mapped to the reference sequence without quality-based trimming.

Nucleotide and protein sequences were compared using MAFFT alignment within Geneious R10.0.5.

### Construction of genetically modified strains

Deletion mutants (Supplementary Table S7) were constructed according to the method described by Datsenko and Wanner^55^ using the primer sets listed in Supplementary Table S6 to amplify a FRT-flanked-CmR-gene with homology extensions to the respective gene region to be deleted. After selecting the Cm^R^-positive deletion mutants, the Cm^R^ gene was eliminated using the helper plasmid pCP20^55^. After the Cm^R^-gene was eliminated, the strains were tested for the correct gene deletion by Sanger sequencing using the primer sets indicated in Supplementary Table S6. To transfer the pAFP_12-05829_ plasmid into a recipient strain, a transposase located on the plasmid was replaced by a Cm^R^ gene as mentioned above. The pAFP_12-05829_ Δ*transposase*::CmR plasmid was purified and introduced into the K12 C600 recipient strain by electroporation with a Life Technologies Cell-Porator according to the manufacturer’s specifications as previously described^56^. After elimination of the Cm^R^ gene, the *afp* deletion was introduced, resulting in K12 C600 (pAFP_12-05829_ Δ*transposase* Δ*afp*::Cm^R^). To complement the *afpA* deletion, the *afp* operon of strain 12-05829 was cloned into pBeloBac11 via Gibson Assembly using the primer set indicated in Supplementary Table S6 ^57^.

### PCR for the detection of afpB, afpD, afpP, afpA2 and afpR

PCR was performed using primer sets shown in Supplementary Table S6. Each reaction contained 2.5 μl of 10 × PCR buffer (NEB, Germany), 1 unit of Taq DNA polymerase (NEB, Germany), 5 pmol of each forward and reverse primer, 200 μM of each deoxynucleoside triphosphate (Bioline, Germany) and distilled water to a total reaction volume of 15 μl. A small amount of a single bacterial colony resuspended in 10 µl of distilled water and heated for 10 min at 95 °C or 10 ng plasmid DNA was used as DNA template. DNA amplification was performed in a PCR thermal cycler using the following conditions: 94 °C for 5 min, followed by 30 cycles of 30 seconds at 94 °C, 1 min at 55 °C, and 1 min at 72 °C, with a final extension of 5 min at 72 °C.

### SDS-PAGE

Ten microliters of an overnight culture of *E. coli* was incubated in 1 ml of DMEM (with 0.45% glucose, Lonza, Germany) containing 1% mannose at 37 °C and 5% CO_2_ for 4 h. Subsequently, the cells were pelleted, lysed in 75 µl of BugBuster (Novagen, Germany) plus 25 µl of 4× Laemmli Buffer, and heated to 95 °C for 10 min. Fifteen microliters of each sample was loaded onto a 15% SDS-polyacrylamide gel, and Coomassie staining was performed after electrophoresis^58^.

### Bacterial autoaggregation assay

For the autoaggregation assay, bacteria were grown overnight in LB and then were inoculated 1:100 into 1 ml of DMEM (with 0.45% glucose, Lonza, Germany) containing 1% mannose in a 24-well plate and incubated for 3 h statically at 37 °C. Light microscopy was performed to detect the autoaggregation phenotype using a Nikon Eclipse inverted microscope (Nikon Instruments, Germany) at 200-fold magnification. For higher resolution, aggregates were analyzed by scanning electron microscopy (SEM). Round glass coverslips (12 mm, Thomas Scientific, Germany) were placed into the wells of a 24-well plate and autoaggregation was assessed as described above. K12 C600 bacterial cells were applied to plastic culture dishes (IBIDI dish; µ-Dish 35 mm, high, IBIDI, Germany) for aggregation assay in a volume of 3 ml. At the end of the aggregation assay, bacteria were fixed in 1% paraformaldehyde, 2.5% glutardialdehyde, 0.05 M HEPES buffer (pH 7.2) for 2 h at room temperature and then were gently washed with distilled water prior to postfixation in 1% OsO_4_ (1 h). Samples were again washed in distilled water, dehydrated in an ethanol series (30, 50, 70, 90 and 96% for 15 min each and absolute ethanol for 30 min) and then were critical point dried (CPD 300, Leica, Germany) using carbon dioxide. Finally, the samples were coated with 3 nm gold/palladium using a sputter coater (E5100 Polaron/Quorum Technologies, UK) and examined using a field emission SEM (Leo 1530 Gemini, Carl Zeiss Microscopy, Germany) with a 5 kV acceleration voltage and a working distance of 4.2 mm. Signals from an in-lens-SE and an Everhart-Thornley secondary electron detector were merged (50:50) for all samples.

### Adherence to HEp-2 cells

HEp-2 epithelial cells were grown to 70–90% optical confluence in 24-well plates on glass coverslips (8 mm, Thomas Scientific, Germany) in DMEM FCS (with 0.45% glucose and 10% FCS; Lonza, Germany). Before adding bacteria, HEp-2 cells were washed three times with DMEM. Bacteria were grown overnight in LB broth, and 10 µl (~MOI of 100) were added to the HEp-2 cells with 1 ml DMEM containing 1% mannose and incubated for 3 h statically at 37 °C with 5% CO_2_. HEp-2 cells were washed five times with PBS, followed by fixation with 3% PFA for 20 min at room temperature. Samples were subsequently air dried and mounted for phase contrast microscopy at 1000-fold magnification on a Nikon Eclipse inverted microscope. For the quantitative HEp-2 adherence assay, 1 ml of PBS and 10 µl of 10% saponin was added after washing with PBS, followed by pipetting up and down approximately ten times, stepwise dilution in PBS and plating on LB agar to determine colony forming units (CFU). As reference for the amount of bacteria multiplied within the 3 h, the washing step with PBS was omitted and the counted CFUs was set to 100%.

### Cytotoxicity assay

HEp-2 epithelial cells were grown to 70–90% optical confluence in 96-well plates in DMEM FCS (with 0.45% glucose and 10% FCS; Lonza, Germany). HEp-2 cells were washed three times with DMEM and then inoculated with bacteria cultured overnight (1:100) in DMEM containing 1% mannose (6 wells per strain). After 24 h, 50 µl of the supernatant was used with the CytoTox 96® Non-Radioactive Cytotoxicity Assay (Promega, Germany) to measure the relative cytotoxicity due to the release of lactate dehydrogenase (LDH) by leaky HEp-2 cells. Lysed HEp-2 cells without the addition of bacteria represented the maximum LDH release (100% cytotoxicity) according to manufacturer’s instructions.

## Supplementary information

Figure_S1

Figure_S2

Table S1

Table S2

Table S3

Table S4

Table S5

Table S6

Table S7
