# Supplementary material for: Novel type of pilus associated with a Shiga-toxigenic E. coli hybrid pathovar conveys aggregative adherence and bacterial virulence
Source: Emerg Microbes Infect. 2018 Dec 5;7:203. doi: 10.1038/s41426-018-0209-8 (PMC6279748; doi:10.1038/s41426-018-0209-8)

Figure S1: *pil* operon mutant of Shiga-toxigenic hybrid *E. coli* 12-05829 does not show adherence defects. Depicted are a quantitative adhesion assay (A) and light microscopy images with 200fold magnification of aggregative adherence to HEp-2 epithelial cells (B). Strain K12c600 without aggregative adherence was used as negative control.

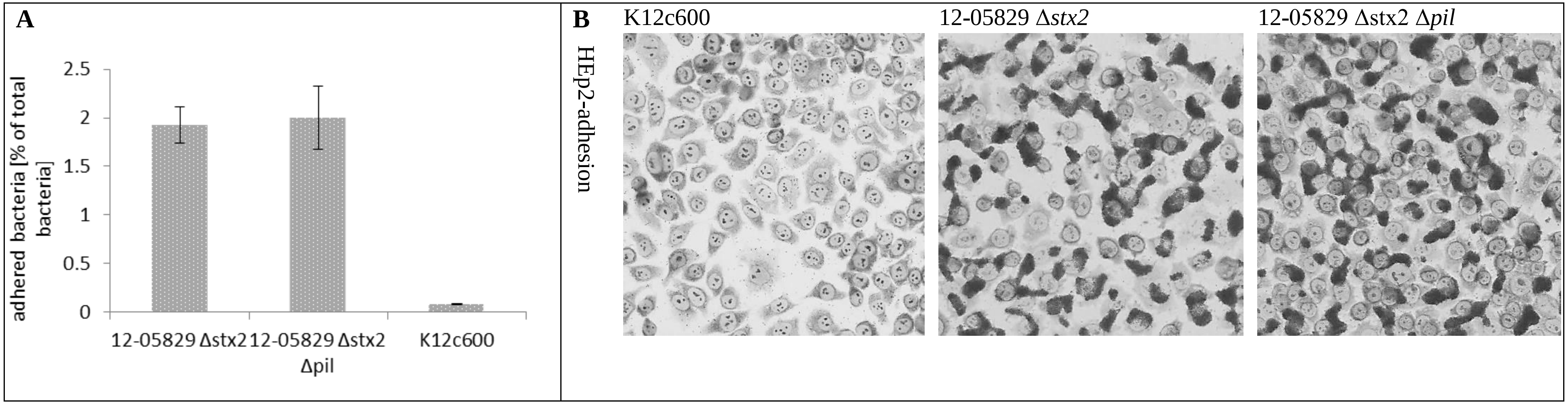

Supplement: Supplementary file 1 — Figure_S1 [file 41426_2018_209_MOESM1_ESM.pdf]
