# Supplementary material for: Novel type of pilus associated with a Shiga-toxigenic E. coli hybrid pathovar conveys aggregative adherence and bacterial virulence
Source: Emerg Microbes Infect. 2018 Dec 5;7:203. doi: 10.1038/s41426-018-0209-8 (PMC6279748; doi:10.1038/s41426-018-0209-8)

Figure S2: Detailed view of the aggregate-forming pili of EHEC/ EAEC strain 12-05829 Δ*stx2*. Depicted is a scanning electron microscopy image with 150 000 fold magnification.

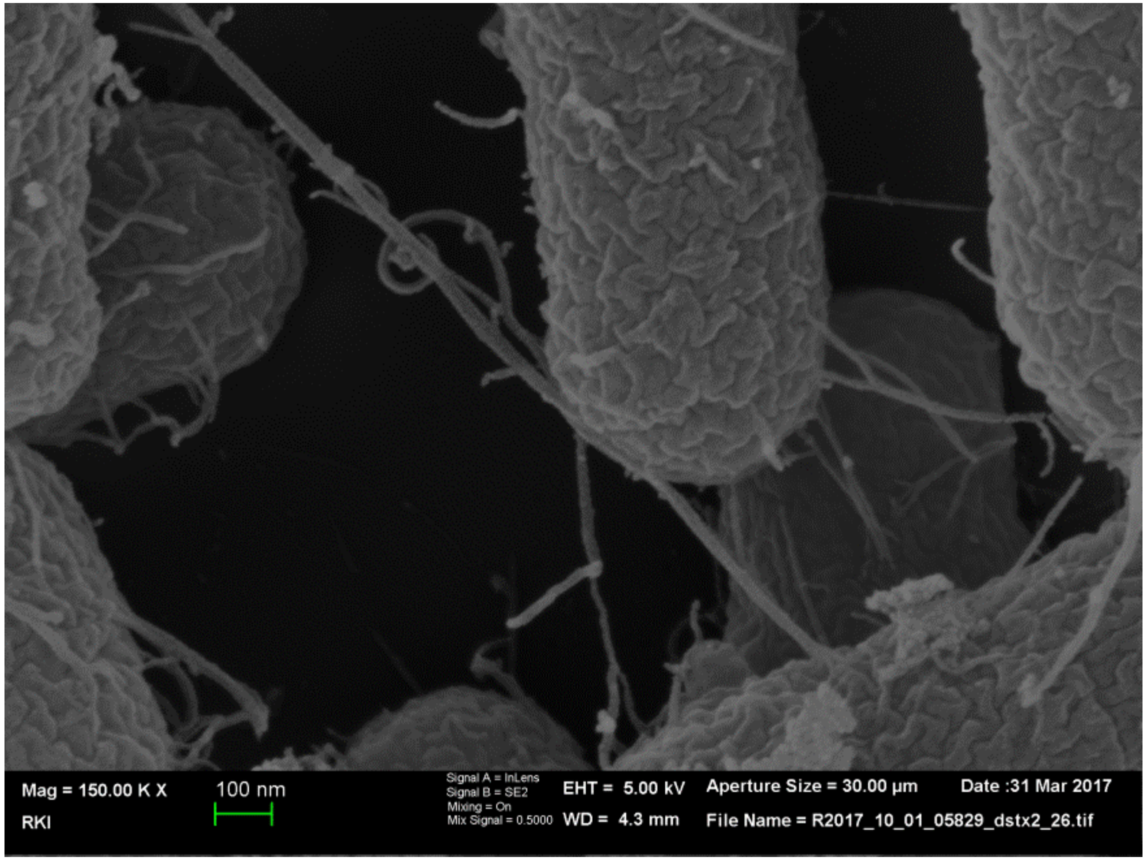

Supplement: Supplementary file 2 — Figure_S2 [file 41426_2018_209_MOESM2_ESM.pdf]
