# Supplementary material for: Novel type of pilus associated with a Shiga-toxigenic E. coli hybrid pathovar conveys aggregative adherence and bacterial virulence
Source: Emerg Microbes Infect. 2018 Dec 5;7:203. doi: 10.1038/s41426-018-0209-8 (PMC6279748; doi:10.1038/s41426-018-0209-8)
Supplement: Supplementary file 3 — Table S1 [file 41426_2018_209_MOESM3_ESM.pdf]

Table S1: Gene or encoded protein identity of Shiga toxigenic hybrid EHEC/EAEC 12-05829 *afp* compared to EPEC *bfp* (GenBank: NC_011603.1) *bfp* and prediction of putative protein functions.

Name	Putative protein functions	Conserved domains	DNA identity to pMAR2 *bfp* [%]	Protein identity to pMAR2 Bfps [%]
**AfpA**	major structural subunit of bundle-forming pilus	bundulin, PilS	50.81	44.18
**AfpG**	hypothetical protein	toxin co-regulated pilus biosynthesis protein Q	43.80	28.02
**AfpB**	membrane protein	Secretin	51.14	44.43
**AfpC**	hypothetical protein	none	46.55	30.50
**AfpU**	hypothetical protein	none	39.72	22.80
**AfpD**	DNA binding protein; type II/IV SS family protein	P-loop NTPase	57.23	55.97
**AfpE**	type II SS protein F	Type II secretory pathway, PulF	61.80	60.23
**AfpF**	type II/IV SS family protein	P-loop NTPase	58.43	59.20
**AfpP**	prepilin peptidase	Peptidase A24	59.18	50.79
**AfpH**	lytic transglycosylase	lysozyme like	58.40	57.93
**AfpI**	hypothetical protein	none	39.50	20.63
**AfpJ**	hypothetical protein	none	41.96	23.50
**AfpK**	hypothetical protein	none	43.10	22.82
**AfpL**	hypothetical protein	none	36.31	17.61
**AfpA$_2$**	major structural subunit of bundle-forming pilus	bundulin, PilS	52.83*	42.71*
