# Supplementary material for: Novel type of pilus associated with a Shiga-toxigenic E. coli hybrid pathovar conveys aggregative adherence and bacterial virulence
Source: Emerg Microbes Infect. 2018 Dec 5;7:203. doi: 10.1038/s41426-018-0209-8 (PMC6279748; doi:10.1038/s41426-018-0209-8)
Supplement: Supplementary file 4 — Table S2 [file 41426_2018_209_MOESM4_ESM.pdf]

Table S2: Nucleotide alignment identity [in %] (A) and protein identity [in %] (B) of *bfp* EPEC operons with *afp* operon of strain 12-05829. (NCBI Accession: [a]AB024946; [b]JHSI01000087; [c]FM180569; [d]JHTJ01000106.1; [e]JHRJ01000140)

**A**	*bfp*_pB171	*bfp*-pEAF_401091	*bfp*_pMAR2	*bfp*-pEAF_403116	*bfp*-pEAF_303289	*afp*-12-05829
*bfp*_pB171[a]		99.96	99.88	99.87	99.02	52.28
bfp-pEAF_401091[b]	99.96		99.91	99.9	99.03	52.27
*bfp*_pMAR2[c]	99.88	99.91		99.96	98.97	52.24
bfp-pEAF_403116[d]	99.87	99.9	99.96		98.96	52.26
bfp-pEAF_303289[e]	99.02	99.03	98.97	98.96		52.31
afp-12-05829	52.28	52.27	52.24	52.26	52.31	

**B**	BFP_pB171	BFP-pEAF_401091	BFP_pMAR2	BFP-pEAF_403116	BFP-pEAF_303289	AFP-12-05829
BFP_pB171[a]		99.87	99.87	99.89	98.96	42.17
BFP-pEAF_401091[b]	99.87		99.79	99.81	98.82	42.14
BFP_pMAR2[c]	99.87	99.79		99.92	98.82	42.19
BFP-pEAF_403116[d]	99.89	99.81	99.92		98.85	42.19
BFP-pEAF_303289[e]	98.96	98.82	98.82	98.85		42.17
AFP-12-05829	42.17	42.14	42.19	42.19	42.17	
