# Supplementary material for: Novel type of pilus associated with a Shiga-toxigenic E. coli hybrid pathovar conveys aggregative adherence and bacterial virulence
Source: Emerg Microbes Infect. 2018 Dec 5;7:203. doi: 10.1038/s41426-018-0209-8 (PMC6279748; doi:10.1038/s41426-018-0209-8)
Supplement: Supplementary file 5 — Table S3 [file 41426_2018_209_MOESM5_ESM.pdf]

Table S3: Strain sequences used for the study available at NCBI

E. coli strain	Serotype	country	collection date	host	isolation source	Accession	pathovar	*ST*	*aat*	*aap*	*aggR*	*AAF*	*stx*	*eae*	*hlyA*	*bfp*	*bfpT*	*afp*	*afpR*	Reference
2-005-03_S4_C1	O?:H10	Tanzania	2009	human	stool	NZ_JNPS00000000	EAEC/ *afp*	43	+	+	-	-	-	-	-	-	-	+	+	Silbergeld et al. 2014, unpublished
2-011-08_S3_C1	O?:H10	Tanzania	2009	human	stool	NZ_JNNB00000000	EAEC/ *afp*	10	+	+	-	-	-	-	-	-	-	+	+	
2-011-08_S3_C2	O111:H12	Tanzania	2009	human	stool	NZ_JMGU00000000	EAEC/ *afp*	43	+	+	-	-	-	-	-	-	-	+	+	
2-011-08_S3_C3	O?:H10	Tanzania	2009	human	stool	NZ_JMGV00000000	EAEC/ *afp*	10	+	+	-	-	-	-	-	-	-	+	+	
2-316-03_S4_C2	O?:H10	Tanzania	2009	human	stool	NZ_JNQT00000000	EAEC/ *afp*	10	+	+	-	-	-	-	-	-	-	+	+	
2-460-02_S3_C1	O111:H12	Tanzania	2009	human	stool	NZ_JNRB00000000	EAEC/ *afp*	3281	+	+	-	-	-	-	-	-	-	+	+	
2-460-02_S3_C2	O111:H12	Tanzania	2009	human	stool	NZ_JNRC00000000	EAEC/ *afp*	3281	+	+	-	-	-	-	-	-	-	+	+	
2-474-04_S3_C1	O?:H10	Tanzania	2009	human	stool	NZ_JNRH00000000	EAEC/ *afp*	43	+	+	-	-	-	-	-	-	-	+	+	
2-474-04_S3_C2	O?:H10	Tanzania	2009	human	stool	NZ_JNRI00000000	EAEC/ *afp*	43	+	+	-	-	-	-	-	-	-	+	+	
2-474-04_S3_C3	O?:H10	Tanzania	2009	human	stool	NZ_JNRL00000000	EAEC/ *afp*	43	+	+	-	-	-	-	-	-	-	+	+	
3-073-06_S3_C2	O?:H10	Tanzania	2009	human	stool	NZ_JNRX00000000	EAEC/ *afp*	43	+	+	-	-	-	-	-	-	-	+	+	
7-233-03_S3_C1	O?:H10	Tanzania	2009	human	stool	NZ_JORO00000000	EAEC/ *afp*	10	+	+	-	-	-	-	-	-	-	+	+	
7-233-03_S3_C3	O?:H10	Tanzania	2009	human	stool	NZ_JORV00000000	EAEC/ *afp*	10	+	+	-	-	-	-	-	-	-	+	+	
381-3	O126:H2	Netherland	2013	human	stool	NZ_JRLM00000000	EAEC/ *afp*	10	+	+	-	-	-	-	-	-	-	+	+	Zhou at al. 2015, CMI
CS01	O?:H30	USA	2013	human	stool	JNOE00000000	EAEC/ *afp*	?	+	+	-	-	-	-	-	-	-	+	+	Garrett et al. 2014, unpublished
MRE600	O150:H9	UK	1950	?	?	CP014197	EAEC/ *afp*	?	+	+	-	-	-	-	-	-	-	+	+	Kurylo et al. 2016, Genome Biol Evol
O104:H4 str. 2011C-3493	O104:H4	USA	2011	human	stool, HUS, Germany stay	CP003289	EHEC/ EAEC	678	+	+	+	I	2	-	-	-	-	-	-	Ahmed et al. 2012. PLOS One
0221_13	O20:H19	UK	2013	human	stool	SAMN02730204	EAEC	278	+	+	+	IV	-	-	-	-	-	-	-	Dallman et al. 2014 PLoS One
0217_13	O33:H16	UK	2013	human	stool	SAMN02730209	EAEC	295	+	+	+	II	-	-	-	-	-	-	-	
1065_13	O63:H12	UK	2013	human	stool	SAMN02730207	EAEC	1664	+	+	+	III	-	-	-	-	-	-	-	
0222_13	O19:H30	UK	2013	human	stool	SAMN02730205	EAEC	38	+	+	+	III	-	-	-	-	-	-	-	
0214_13	OX:H19	UK	2013	human	stool	SAMN02730208	EAEC	5601	+	+	+	III	-	-	-	-	-	-	-	
1061_13	O55:H19	UK	2013	human	stool	SAMN02730206	EAEC	10	+	+	+	III	-	-	-	-	-	-	-	
0220_13	O131:H27	UK	2013	human	stool	SAMN02730198	EAEC	5533	+	+	+	I	-	-	-	-	-	-	-	
O26:H11 str. 11368	O26:H11	Japan	2001	human	stool, diarrhea	NC_013361	EHEC	21	-	-	-	-	1	+	+	-	-	-	-	Ogura et al. 2009, Proc Natl Acad Sci USA
O157:H7 str. EDL933	O157:H7	USA	1982	beef	food	NZ_CP008957	EHEC	11	-	-	-	-	1,2	+	+	-	-	-	-	Latif et al. 2014, Gen Announc
O103:H2 str. 12009	O103:H2	Japan	2001	human	stool, diarrhea	NC_013353	EHEC	17	-	-	-	-	1,2	+	+	-	-	-	-	Ogura et al. 2009, PNAS
0127:H6 str. E2348/69	0127:H6	UK	1969	human	stool, diarrhea	NC_011601	EPEC	15	-	-	-	-	-	+	-	+	+	-	-	Iguchi et al. 2009, J Bac
Ec 042	O44:H18	Peru	1983	human	stool, diarrhea	NC_017626	EAEC	414	+	+	+	II	-	-	-	-	-	-	-	Chaudhuri et al. 2010, PLoS ONE
K-12 substr. MG1655	O16:H48	US	1922	human	stool	NC_000913	apathogen	10	-	-	-	-	-	-	-	-	-	-	-	Hayashi et al. 2006, Mol Syst Biol
