# Supplementary material for: Novel type of pilus associated with a Shiga-toxigenic E. coli hybrid pathovar conveys aggregative adherence and bacterial virulence
Source: Emerg Microbes Infect. 2018 Dec 5;7:203. doi: 10.1038/s41426-018-0209-8 (PMC6279748; doi:10.1038/s41426-018-0209-8)
Supplement: Supplementary file 6 — Table S4 [file 41426_2018_209_MOESM6_ESM.pdf]

Table S4: Nucleotide alignment identity [in %] of *afp* operons found in NCBI by BLAST analysis with the *afp* operons of strain 12-05829 and 12-05898

	Nucleotide identity to $afp_{12\text{-}05829}$ [%]
$afp_{12\text{-}05898}$	98.567
afp_{DEC6C}	98.286
$afp_{Ec\_2\text{-}005\text{-}03\_S4\_C1}$	97.675
$afp_{Ec\_2\text{-}011\text{-}08\_S3\_C1}$	96.301
$afp_{Ec\_2\text{-}011\text{-}08\_S3\_C2}$	97.683
$afp_{Ec\_2\text{-}011\text{-}08\_S3\_C3}$	96.309
$afp_{Ec\_2\text{-}316\text{-}03\_S4\_C2}$	95.684
$afp_{Ec\_2\text{-}460\text{-}02\_S3\_C1}$	98.328
$afp_{Ec\_2\text{-}460\text{-}02\_S3\_C2}$	98.345
$afp_{Ec\_2\text{-}474\text{-}04\_S3\_C1}$	97.683
$afp_{Ec\_2\text{-}474\text{-}04\_S3\_C2}$	97.674
$afp_{Ec\_2\text{-}474\text{-}04\_S3\_C3}$	97.683
$afp_{Ec\_3\text{-}073\text{-}06\_S3\_C2}$	97.674
$afp_{Ec\_7\text{-}233\text{-}03\_S3\_C1}$	96.237
$afp_{Ec\_7\text{-}233\text{-}03\_S3\_C3}$	96.237
$afp_{Ec\_381\text{-}3}$	95.603
afp_{Ec_CS01}	97.458
afp_{MRE600}	97.816
