# Supplementary material for: Novel type of pilus associated with a Shiga-toxigenic E. coli hybrid pathovar conveys aggregative adherence and bacterial virulence
Source: Emerg Microbes Infect. 2018 Dec 5;7:203. doi: 10.1038/s41426-018-0209-8 (PMC6279748; doi:10.1038/s41426-018-0209-8)
Supplement: Supplementary file 7 — Table S5 [file 41426_2018_209_MOESM7_ESM.pdf]

Table S5: Further *aatA*-positive, AAF/I-V-negative *E. coli* strains from collection of German National Reference Centre for *Salmonella* and other enteric bacterial pathogens used in this study.

	Strain No.	Phenotypic Serovar	Pathovar	Material	Clinical symptoms	MLST ST	*afp* operon*	*afpR*	auto-aggregation	References
aatA positive / AAF I-V negative	01-05171	O130:H27	EAEC	human, stool	n.s.	130	-	-	-	this study
	03-08481	O78:H10	EAEC	human, stool	diarrhea	10	-	-	-	this study
	04-06501	O111:H12	EAEC	n.s.	n.s.	10	+	+	+	this study
	05-03519	O98:H-	EAEC	human, stool	n.s.	306	-	-	-	this study
	05-05882	O128:H35	EAEC	human, stool	n.s.	1147	+	+	+	this study
	05-07641	O103:H43	EAEC	human, stool	n.s.	218	+	+	+	this study
	06-02737	O128:H35	EAEC	human, stool	n.s.	1147	+	+	+	This study
	06-05401	O78:H10	EAEC	human, stool	bloody stool	10	-	-	-	this study
	06-08668	Ont:H-	EAEC	human, stool	n.s.	34	+	+	+	this study
	07-02828	Orauh:H-	EAEC	human, stool	diarrhea	34	+	+	+	this study
	07-06796	Orauh:H-	EAEC	human, stool	diarrhea	10	+	+	+	this study
	07-06883	O103:H43	EAEC	human, stool	n.s.	218	+	+	-	this study
	08-04199	O114:H10	EAEC	human, stool	diarrhea	10	-	-	-	this study
	08-04688	Ont:H-	EAEC	human, stool	n.s.	34	+	+	-	this study
	09-02242	O128:H35	EAEC	human, stool	diarrhea	1147	+	+	+	this study
	10-06624	Orauh:H-	EAEC	human, stool	n.s.	206	+	+	+	this study
	10-06632	O142:Hnt	EAEC	human, stool	n.s.	2706	+	+	+	this study
	10-06641	O128:H35	EAEC	human, stool	n.s.	1147	+	+	+	this study
	10-06642	Ont:H-	EAEC	human, stool	n.s.	34	+	+	+	this study
	10-06644	O130:H27	EAEC	human, stool	n.s.	130	-	-	-	this study
	11-07838	Ont:Hnt	EAEC	human, stool	n.s.	10	+	+	+	this study
	12-01549	O111:H-	EAEC	human, stool	diarrhea	3012	-	-	-	this study
	12-03007	Orauh:H-	EAEC	human, stool	diarrhea	3281	+	+	+	this study
	12-03634	O55:H25	EAEC	human, stool	n.s.	1114	+	+	+	this study
	12-03637	Orauh:H-	EAEC	human, stool	diarrhea	10	+	+	+	this study
	12-05897	O130:H27	EAEC	human, stool	diarrhea	31	-	-	-	this study
	13-03827	O128:H-	EAEC	human, stool	n.s.	1147	+	+	+	this study
	13-04709	O128:H-	EAEC	human, stool	diarrhea	10	+	+	+	this study
	13-05154	Orauh:H-	EAEC	human, stool	n.s.	10	-	-	-	this study
	14-01249-2	Ont:H-	EAEC	human, stool	n.s.	34	+	+	+	this study
	14-01687	O78:H-	EAEC	human, stool	n.s.	34	+	+	+	this study
	14-02904	O55:H27	EAEC	human, stool	diarrhea	495	+	+	+	this study
	14-05802-2	Ont:H10	EAEC	human, stool	diarrhea	43	+	+	+	this study

* tested for *afpA2, B, D* and *P* genes (*afp* operon markers) by means of PCR. . n.s.=not specified, +=positive, -=negative
