# Supplementary material for: Novel type of pilus associated with a Shiga-toxigenic E. coli hybrid pathovar conveys aggregative adherence and bacterial virulence
Source: Emerg Microbes Infect. 2018 Dec 5;7:203. doi: 10.1038/s41426-018-0209-8 (PMC6279748; doi:10.1038/s41426-018-0209-8)
Supplement: Supplementary file 8 — Table S6 [file 41426_2018_209_MOESM8_ESM.pdf]

Table S6: Primers used in this study

purpose	Name	Sequence [3´→5`]	Template
PCR product for *stx2*-deletion	stx2_del_up	ATGAAGTGTATATTGTTAAAATGGGTGCTGTGCCTGTTACGTGTAGGCTGGAGCTGCTTC	pKD3
	stx2_del_down	TTAATTAAACTGCACTTCAGCAAATCCTGAACCTGACGCACATATGAATATCCTCCTTAG	
PCR product for *afp*-deletion	afp_del_up	ATCTAAACTGATTTCTACATAACAAAAGTGTGTCAGCGAAGTGTAGGCTGGAGCTGCTTC	pKD3
	afp_del_down	AAATCTCCACCTTGTGGGAGACCAGCGTCATTACTAGCCGCATATGAATATCCTCCTTAG	
PCR product for *afpA*$_{12\text{-}05829}$-deletion	afpA$_{29}$_del_up	TTGATTTGGTTGTTTGTTGATTTAAATCAAAATCCAAACGTGTAGGCTGGAGCTGCTTC	pKD3
	afpA$_{29}$-del_down	TTATTTCAGCAGGAAGGCGATTTGGTTGGGGCCTGTTTTCATATGAATATCCTCCTTAG	
PCR product for *afpA*$_{12\text{-}05898}$-deletion	afpA$_{98}$_del_up	TTGATTTGGCTATTGGTTTATTTAAATCAAAATCCAAACGTGTAGGCTGGAGCTGCTTC	pKD3
	afpA$_{98}$_del_down	TTATTTCAGCAGGAAAGCGATCTTGTTGGTGTTTTTTTTCATATGAATATCCTCCTTAG	
PCR product for *afpA2*-deletion	afpA$_2$_del_up	ATGGTTTTTAATGTATTTTATATGAGGATACTATAATATTGTGTAGGCTGGAGCTGCTTC	pKD3
	afpA$_2$_del_down	AAATCTCCACCTTGTGGGAGACCAGCGTCATTACTAGCCGCATATGAATATCCTCCTTAG	
PCR product for *afpR*-deletion	afpR_del_up	CTAATTATGAAGCTGATAATAATGTGTCGTTATTACGTGTAGGCTGGAGCTGCTTC	pKD3
	afpR_del_down	TTGGAGTTACTGTATTAAAATGAATTATACCATCTACATATGAATATCCTCCTTAG	
PCR product for transposase-deletion	Trans_del_up	TCACTTCCTGACGATATCAATGCACTGAAACGTCTCGTGTAGGCTGGAGCTGCTTC	pKD3
	Trans_del_down	CCTCCATGATCCGAACCGAAGAACAGGTAGTTTTTGCATATGAATATCCTCCTTAG	
PCR product for *pil*-operon deletion	pil_del_fw	ATGCAAAAAAAAATCACTTACACTCCTTCTGCCGTCATTACGTGTAGGCTGGAGCTGCTTC	pKD3
	pil_del_rv	TCAGCTTGCTTTGACGATGTAGTTAAAGGCAATGTTTTTGCATATGAATATCCTCCTTAG	
Proof of *afp*-deletion	afp_fw	ACTACTTAGGCAGAGGGGCA	pAFP$_{12\text{-}05829}$ Δ*afp*
	afp_rv	ACTGCCATCCCAGAATGGTG	
Proof of *afpA*-deletion	afp_fw	ACTACTTAGGCAGAGGGGCA	pAFP$_{12\text{-}05829}$ Δ*afpA*
	afpA_G_rv	ACGGAAAAGCCAGCATAAGC	
Proof of *afpA2*-deletion	afpK_fw	CCTGTTGTATATTTCAAACTGGAACA	pAFP$_{12\text{-}05829}$ Δ*afpA$_2$*
	*afp*_rv	ACTGCCATCCCAGAATGGTG	
Proof of *afpR*-deletion	afpR_fw	CAAAAACGGGTGGCGTACTG	pAFP$_{12\text{-}05829}$ Δ*afpR*
	afpR_rv	ACCTGAGCAGACTGACAACG	
Proof of transposase-deletion	transpos_fw	GCAGCTCACTTCCTGACGAT	pAFP$_{12\text{-}05829}$ Δtransposase
	transpos_rv	CTCCTCCATGATCCGAACCG	
Proof of *pil*-operon deletion	pil_fw	ATCCAGAAACCGCGTTCAGT	pHly12-05829 Δ*pil*
	pil_rv	ATTCGGCCTGTTATGACGCA	
Cloning pBeloBac11 *afp*$_{12\text{-}05829}$ →Gibson assembly with 3 PCR products	pBeloB_part1_rv	TGCCCCTCTGCCTAAGTAGTCATGCCTGCAGGTCGACTCTAGAGG	pBeloBac11 and pAFP$_{12\text{-}05829}$
	pBeloB_part2_fw	CACCATTCTGGGATGGCAGTCATGCAAGCTTGAGTATTCTATAGT	
	afp_p1_extp2_rv	CTAAATCAGGATCGATCCGTAAGCTTAAAATCATCAACAC	
	afp_p1_extV_fw	CCTCTAGAGTCGACCTGCAGGCATGACTACTTAGGCAGAGGGGCA	
	afp_p2_extp1_fw	AAGCTTACGGATCGATCCTGATTTAGTAACAGGTCTGA	
	afp_p2_extV_rv	ACTATAGAATACTCAAGCTTGCATGACTGCCATCCCAGAATGGTG	
detection of *afpA*$_2$	afpA2_fw	ATCGTCTCCGCTGTTAACGG	Colony suspension and plasmid DNA
	afpA2_rv	TTTACAAGCATTGGCAGCACCAGC	
detection of *afpB*	afpB_fw	TACAACGATGTCAGGGGTGC	
	afpB_rv	TTACCCTGTCGCTTTCCACC	
detection *afpD*	afpD_fw	AGGAAGAACGAGGGAGGGAA	
	afpD_rv	CCCAGTGGTCAACAGCTTCT	
detection *afpP*	afpP_fw	ATATGGTCGCCACCTTCGAG	
	afpP_rv	TCAATGAGGGGCCCATAGGA	
detection *afpR*	afpR_det_fw	GTGAAGAACATTATTGAAGGGGGC	
	afpR_det_rv	CATCACTTAATCGCCAGCGTT	
verification plasmid ring closure	12-05829_Pl1_fw	GTCACTGACGAGAACACCGT	Plasmid DNA of strain 12-05829
	12-05829_Pl1_rv	GTCACCAAAAACCTGACGCC	
	12-05829_Pl2_fw	CTGTCAGCAGCGTCTTCTGA	
	12-05829_Pl2_rv	TGTTCGTGTACTGGACGGTG	
	12-05829_Pl3_fw	TATTGGATGGGGGCTTGCAG	
	12-05829_Pl3_rv	AACGTCCATTGAAGACGCCT	
