# Supplementary material for: Novel type of pilus associated with a Shiga-toxigenic E. coli hybrid pathovar conveys aggregative adherence and bacterial virulence
Source: Emerg Microbes Infect. 2018 Dec 5;7:203. doi: 10.1038/s41426-018-0209-8 (PMC6279748; doi:10.1038/s41426-018-0209-8)
Supplement: Supplementary file 9 — Table S7 [file 41426_2018_209_MOESM9_ESM.pdf]

Table S7: Genetically modified strains

Strain background	Genetic modification	remark
**EHEC/EAEC 12-05829**	*Δstx2*	deletion of *stx2* gene
	Δstx2 Δafp operon	deletion of whole *afp* operon
	Δstx2 ΔafpA	deletion of major pilin gene
	Δstx2 ΔafpA2	deletion of *afpA* homologue
	Δstx2 ΔafpR	deletion of AraC-like regulator
	Δstx2 ΔafpA (pCL138) = (pBeloBac11+$afp_{12\text{-}05829}$)	*afp* operon complementing strain
**EAEC 12-05898**	*Δafp* operon	deletion of whole *afp* operon
	ΔafpA	deletion of major pilin gene
	ΔafpA2	deletion of *afpA* homologue
	ΔafpR	deletion of AraC-like regulator
	ΔafpA (pCL138)	*afp* operon complementing strain
**non-pathogen *E. coli* K12 c600**	$pAFP_{12\text{-}05829}$ $\Delta transposase::Cm^R$	pAFP from strain 12-05829 with deleted transposase/ Cm^R instead inserted
	$pAFP_{12\text{-}05829}$ $\Delta transposase\ \Delta afp::Cm^R$	pAFP with deleted *afp* operon/ Cm^R instead inserted
